# Role of acyl-coenzyme A: cholesterol transferase 1 (ACAT1) in retinal neovascularization

**DOI:** 10.1186/s12974-023-02700-5

**Published:** 2023-01-23

**Authors:** Syed A. H. Zaidi, Tahira Lemtalsi, Zhimin Xu, Isabella Santana, Porsche Sandow, Leila Labazi, Robert W. Caldwell, Ruth B. Caldwell, Modesto A. Rojas

**Affiliations:** 1 Present Address: Vascular Biology Center, Augusta University, 1460 Laney Walker Blvd, Augusta, GA 30912-2500 USA; 2 Culver Vision Discovery Institute, Augusta University, Augusta, GA USA; 3 Department of Pharmacology and Toxicology, Augusta University, Augusta, GA USA; 4 Department of Cellular Biology and Anatomy, Augusta University, Augusta, GA USA

**Keywords:** Hypoxia, Neovascularization, LDL cholesterol, Cholesterol ester, ACAT1, TREM1, Inflammation, Cytokines, Retinopathy of prematurity, Oxygen-induced retinopathy

## Abstract

**Background:**

We have investigated the efficacy of a new strategy to limit pathological retinal neovascularization (RNV) during ischemic retinopathy by targeting the cholesterol metabolizing enzyme acyl-coenzyme A: cholesterol transferase 1 (ACAT1). Dyslipidemia and cholesterol accumulation have been strongly implicated in promoting subretinal NV. However, little is known about the role of cholesterol metabolism in RNV. Here, we tested the effects of inhibiting ACAT1 on pathological RNV in the mouse model of oxygen-induced retinopathy (OIR).

**Methods:**

In vivo studies used knockout mice that lack the receptor for LDL cholesterol (LDLR^−/−^) and wild-type mice. The wild-type mice were treated with a specific inhibitor of ACAT1, K604 (10 mg/kg, i.p) or vehicle (PBS) during OIR. In vitro studies used human microglia exposed to oxygen–glucose deprivation (OGD) and treated with the ACAT1 inhibitor (1 μM) or PBS.

**Results:**

Analysis of OIR retinas showed that increased expression of inflammatory mediators and pathological RNV were associated with significant increases in expression of the LDLR, increased accumulation of neutral lipids, and formation of toxic levels of cholesterol ester (CE). Deletion of the LDLR completely blocked OIR-induced RNV and significantly reduced the AVA. The OIR-induced increase in CE formation was accompanied by significant increases in expression of ACAT1, VEGF and inflammatory factors (TREM1 and MCSF) (*p* < 0.05). ACAT1 was co-localized with TREM1, MCSF, and macrophage/microglia makers (F4/80 and Iba1) in areas of RNV. Treatment with K604 prevented retinal accumulation of neutral lipids and CE formation, inhibited RNV, and decreased the AVA as compared to controls (*p* < 0.05). The treatment also blocked upregulation of LDLR, ACAT1, TREM1, MCSF, and inflammatory cytokines but did not alter VEGF expression. K604 treatment of microglia cells also blocked the effects of OGD in increasing expression of ACAT1, TREM1, and MCSF without altering VEGF expression.

**Conclusions:**

OIR-induced RNV is closely associated with increases in lipid accumulation and CE formation along with increased expression of LDLR, ACAT1, TREM1, and MCSF. Inhibiting ACAT1 blocked these effects and limited RNV independently of alterations in VEGF expression. This pathway offers a novel strategy to limit vascular injury during ischemic retinopathy.

**Graphical Abstract:**

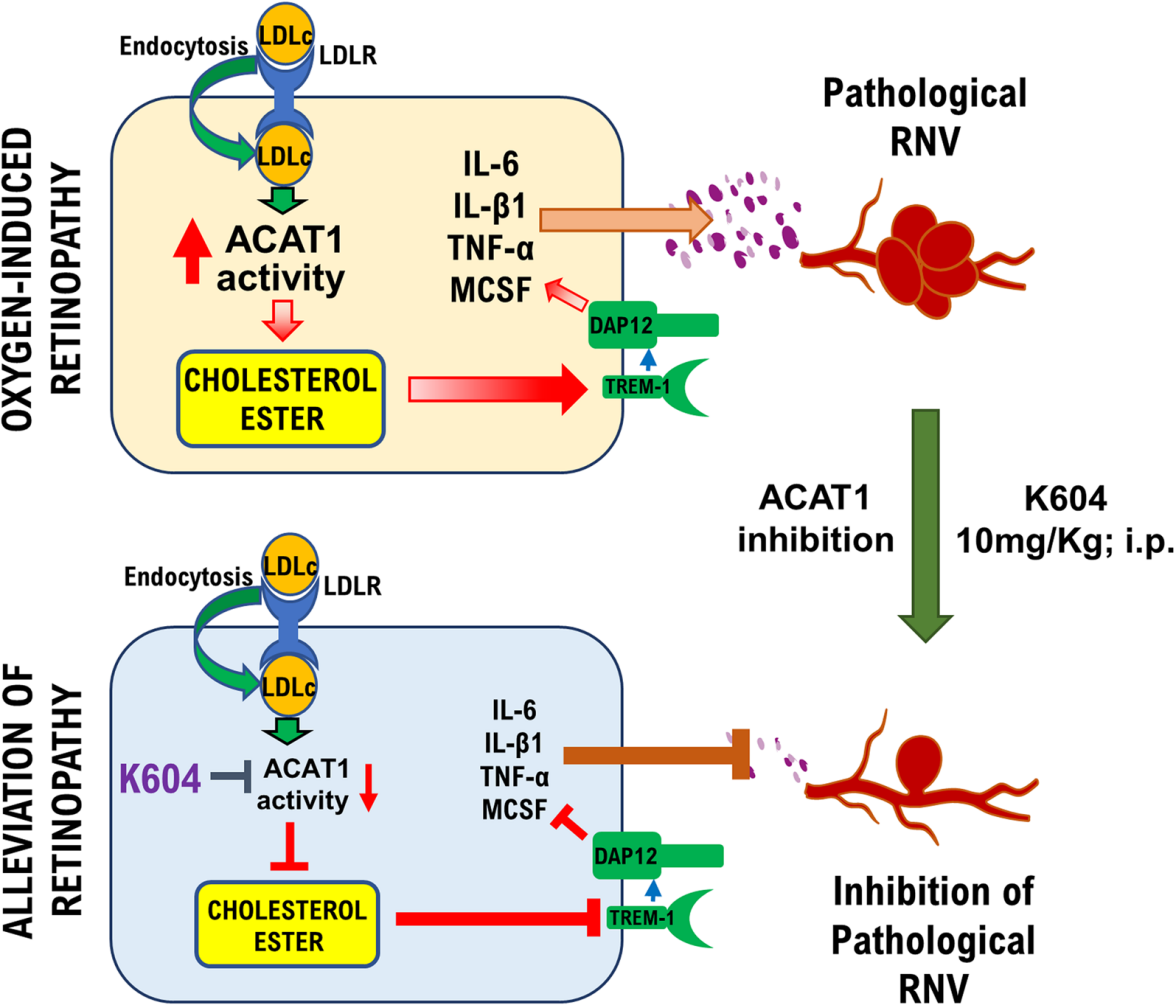

**Supplementary Information:**

The online version contains supplementary material available at 10.1186/s12974-023-02700-5.

## Introduction

Pathological retinal neovascularization (RNV) is a major cause of tissue damage and vision loss in blinding diseases, such as retinopathy of prematurity (ROP) and diabetic retinopathy (DR) [1, 2]. Current therapies, including laser photocoagulation, vitrectomy surgery, and intravitreal injections of vascular endothelial growth factor (VEGF) inhibitors, can limit late-stage vascular pathology, but are not effective for every patient and have some risks of complications. Laser photocoagulation may cause blind spots in areas of scar formation and vitrectomy surgery has some risk of cataract formation, raised intraocular pressure, or infection [3]. Intravitreal injections of anti-VEGF agents have been linked to adverse effects on the photoreceptors and choroidal vessels, as well as on the kidney and cardiovascular system [4, 5]. Thus, there is a great need for a better understanding of the molecular mechanisms underlying RNV and for developing less invasive therapies that can be administered prior to the tissue damage.

We have performed studies using the mouse model of oxygen-induced retinopathy (OIR) to test whether targeting the cholesterol metabolizing enzyme ACAT1 (acyl-Coenzyme A: cholesterol acyltransferase 1) could serve as a novel therapeutic strategy for limiting ischemic retinal injury and pathological RNV. Under normal conditions, excess accumulation of free cholesterol in cells is prevented by its esterification to form cholesterol esters (CE). This reaction is catalyzed by ACAT enzymes, also called sterol *O*-acyltransferases [6]. There are two isoforms of ACAT: ACAT1 and ACAT2 [6, 7]. Both are integral membrane proteins located in the endoplasmic reticulum (ER), and both are allosterically activated by their substrate (cholesterol or oxysterols) [8].

ACAT1 is the major isoform in the retina and is localized mainly to the photoreceptor outer segments. However, ACAT1 deletion does not change the retinal content of CE, suggesting that ACAT1 has a minor role in cholesterol esterification under normal conditions [9]. Whereas cells in peripheral tissues obtain cholesterol mainly by low-density lipoprotein receptor (LDLR)-mediated uptake of circulating cholesterol from the blood [10], cells in the retina like those in the brain, synthesize their own cholesterol [11].

While very little is known about the role of ACAT and CE in RNV, studies in models of subretinal neovascularization have shown a major role of dyslipidemia and cholesterol accumulation in promoting pathological NV [11]. In addition, immunolocalization studies using post-mortem retinas from patients with DR have shown increases in retinal levels of oxidized LDL that correlated with macrophage infiltration and severity of DR [12, 13]. An early report noted the presence of extravascular ApoB and CE, along with increased macrophage infiltration in retinas from DR patients [14]. Recent studies using optical coherence tomography angiography have shown significant increases in the density of “macrophage-like” cells clustered on blood vessels in the retinas of patients with DR as compared with healthy eyes [15]. Studies in a mouse model of early diabetes showed that intravitreal injections of highly oxidized glycated LDL significantly accelerated the progression of retinal injury showing many features of DR [16].

In other diseases there is more knowledge about the involvement of macrophage/microglia and the ACAT1 pathway in the pathology. Increased ACAT1 expression/activity in activated macrophages/microglia has been implicated in inflammatory conditions, such as atherosclerosis, cancer, and Alzheimer’s disease [6, 17, 18]. In models of high fat diet, dyslipidemia and cholesterol accumulation in macrophages has been shown to promote an inflammatory phenotype characterized by increased expression of TREM1 (triggering receptor expressed in myeloid cells1) [19]. TREM1 is a potent amplifier of pro-inflammatory responses in a variety of infectious and non-infectious diseases [20, 21]. It is highly expressed in myeloid cells but is also expressed and inducible in microglia, endothelial cells, and some epithelial cells and plays a direct role in vascular inflammation and dysfunction. TREM1 amplifies inflammatory signaling through its internal adapter DAP12 [22], increasing the expression of MCSF (macrophage colony stimulating factor), VEGF and tumor necrosis factor α (TNFα) [23, 24].

Our previous studies in the mouse model of oxygen-induced retinopathy (OIR) have demonstrated the critical role of TREM1 in pathological RNV [25]. We found that treatment with TREM1 peptide inhibitors almost completely prevented RNV while reducing the area of vaso-obliteration and preventing the OIR-induced increases in expression of MCSF. We have now performed studies to test whether inhibition of the ACAT pathway can limit activation of the TREM1/DAP12 pathway and reduce pathological RNV in the OIR model.

## Materials and methods

### Mice

Experiments were performed using C57BL/6J mice and LDLR^−/−^ mice and their wildtype littermates. The LDLR^−/−^ mice were a generous gift from Dr. Neal Weintraub and were in a C57Bl/6J background (Stock #2207, the Jackson Laboratory). Genotyping was performed according to the Jackson Laboratory protocol.

### Mouse model of OIR

Experiments using mice were performed following the recommendations in the Guide for the Care and Use of Laboratory Animals of the National Institutes of Health and the United States Department of Agriculture Animal Welfare Act (9 CFR, Parts 1, 2, and 3). All experimental procedures were approved by the Institutional Animal Care and Use Committee at Augusta University (Protocol # 2008-0243).

Litters of neonatal C57BL/6J and LDLR^−/−^ mice and nursing dams were maintained in hyperoxia (75% oxygen) from postnatal day 7 (P7) until P12 and then returned to normoxia until P17 and retinas were collected for analysis (Additional file 1: Fig. S1a). One group of wildtype pups was treated with a cinnamic acid derivative (*N*-[3-(4-hydroxyphenyl)-1-oxo-2-propenyl]-l-phenylalanine, methyl ester, Santa Cruz Biotechnology, Dallas, Tx) that inhibits both ACAT1 and ACAT2 isoforms equally [26]. This drug was administered via i.p. injections (10 mg/kg in 50 µL PBS) every 2 days from P7 to P16 and mice were sacrificed on P17 (Additional file 1: Fig. S1b). Additional groups of wildtype pups were treated with daily injections of a specific ACAT1 inhibitor (K604, BioVision, Milpitas CA, 10 mg/kg in 50 µL PBS) from P7 to P16 and sacrificed on P17, from P7 to P11 and sacrificed at P12, or from P12 to P16 and sacrificed on P17 (Additional file 1: Fig. S1b–d). The drug dosage was based on previous reports [27]. Vehicle control groups received PBS alone. Retinas from all groups were collected and prepared for analysis of RNV and vaso-obliteration as well as, expression of TREM1, MCSF, VEGF, ACAT1, and LDLR and levels of cholesterol esters as outlined below.

### Hypoxia exposure in macrophage and microglia cells

Human HMC3 microglia cells were cultured in DMEM medium containing 25 mM glucose, 10% FBS, and 1% P/S. For hypoxia experiments, HMC3 cells were treated with K604 (1 µM) or vehicle in a no glucose DMEM medium and subjected to hypoxia (1% O_2_) for 24 h. For cells undergoing normoxia, media was replaced with serum free DMEM medium containing 25 mM glucose and left in normoxic conditions (21% O_2_). Human THP1 macrophages were cultured in DMEM medium containing 5.5 mM glucose, 10% FBS, and 1% P/S. For hypoxia experiments, THP1 cells were incubated in RPMI medium containing 5.5 mM glucose, 2% FBS, and 1% P/S. Macrophages were treated with K604 (1 µM) or vehicle and subjected to hypoxia (1% O_2_) or normoxia (21% O_2_) for 16 h. Cells were then collected and processed for protein quantification and western blot following our established protocol [25].

### Oil Red O staining for detection of neutral lipids

Cholesterol and other neutral lipids were detected as described previously [28]. Retina frozen sections were reacted with 0.5% Oil Red O (Sigma-Aldrich, St. Louis, MO, dissolved in 1,2-isopropanol, 15 min, room temperature), rinsed in 60% 2-propanol for 5 min and rinsed in distilled water (twice 5 min each time). Sections were mounted in aqueous mounting media to capture the images using a Zeiss Axioplan 2 fluorescence microscope (Carl Zeiss Meditec, Inc., Dublin, CA) equipped with a 420 nm excitation filter, 520 nm barrier filter, and a 20× lens.

### Filipin staining for detection of cholesterol ester (CE)

Retinal frozen sections were reacted with the fluorescent polyene antibiotic filipin (Sigma-Aldrich, St. Louis, MO) to detect CE. This compound binds specifically to sterols and interacts with the 3-β hydroxyl group of cholesterol. For CE detection, we followed the protocol described by Rudolf and Curcio [29]. Native unesterified cholesterol (UC) was extracted from cryosections by rinsing in 60% ethanol for 10 min. Native CE was hydrolyzed with cholesterol esterase (1.65 units/mL) in PBS for 3 h at 37 °C (C9281, Sigma St. Louis, MO). The newly released UC by the hydrolysis of CE was stained with 50 ng/mL filipin in PBS.

### Determination of RNV, AVA and tip cells in retina flat mount

Retinal flat mounts were labeled with Isolectin B_4_ (IB_4_) as described before [30]. Images of the retinal flat mounts were constructed by capturing a series of 12 pictures from all samples using a 5× lens. The images were then regrouped to make a retina map. Next, areas of vaso-obliteration (AVA) and RNV were quantified using NIH ImageJ software as previously described [31]. Retinas flat mount stained with IB_4_ were also used to quantify tip cells by direct observation under fluorescence microscope with a 20× lens. Representative images from each group were taken with a 40× lens. Images were captured with a 20× lens using a Zeiss Axioplan2 fluorescence microscope (Carl Zeiss Meditec, Inc.).

### Immunolocalization

Retinal frozen sections and flat mounts were processed for immunolabeling according to our standard protocol [32]. The samples were blocked with normal goat serum or donkey serum and incubated overnight with IB_4_, anti-TREM1, anti-MCSF1, anti-F4/80, anti-Iba1, anti-ACAT1 or anti-LDLR antibodies. The samples were washed 3 times with PBS and incubated with secondary antibodies (Invitrogen, Waltham, MA). All these antibodies were obtained from various sources, and their catalog information, together with working dilutions, is provided in Additional file 9: Table S1. Tissue sections were also reacted with secondary antibody alone to rule out non-specific reactivity (Additional file 6: Fig. S6). Images were captured using a Zeiss Axioplan2 fluorescence microscope or Zeiss 780 inverted Confocal microscope (Carl Zeiss Meditec, Inc. Dublin, CA).

### Western blotting

Lysates from retina samples and cell lines were prepared for protein quantification and western blot analysis following our established protocol [33]. The samples were homogenized in modified RIPA buffer (20 mM Tris–HCl, 2.5 mM EDTA, 50 mM NaF, 10 mM Na_4_P_2_O_7_, 1% Triton X-100, 0.1% sodium dodecyl sulfate, 1% sodium deoxycholate, 1 mM phenylmethylsulfonylfluoride, pH 7.4). Samples containing equal amounts of protein were separated by 10% or 12% sodium dodecyl sulfate polyacrylamide gel electrophoresis, transferred to polyvinylidene difluoride (PVDF) or nitrocellulose membrane, and reacted for 24 h with anti-LDLR, anti-ACAT1, anti-ACAT2, anti-TREM1, anti-MCSF1, and anti-VEGF antibodies in 2% BSA, followed by incubation with corresponding horseradish peroxidase-linked secondary antibodies (Additional file 7: Fig. S7; Additional file 8: Fig. S8). Bands were quantified by densitometry and the data were analyzed using ImageJ software and normalized to loading control [34]. Equal loading was verified by stripping the membranes and reproving them with a mouse monoclonal antibody against β actin. All these antibodies were obtained from various sources, and their catalog information, together with working dilutions, is provided in Additional file 9: Table S1.

### Quantitative real time RT PCR (qRT PCR) analysis

The total RNA from mouse retina samples was extracted with an RNAqueous 4PCR total RNA isolation kit (Invitrogen, Carlsbad, CA, US), and qRT PCR was performed as described previously [35]. Briefly, a 0.25 μg sample of RNA was utilized as a template for reverse transcription using M-MLV reverse transcriptase (Invitrogen). qRT PCR was performed on an ABI 7500 Real Time PCR System (Applied Biosystems, Foster City, CA) with the respective gene-specific primers listed in Additional file 10: Table S2. The relative gene expression is calculated using the comparative threshold cycle (ΔΔCt) method against the internal control, hypoxanthine phosphoribosyl-transferase (HPRT). Expression levels for all genes are reported as fold change to room air controls.

### Measurement of cholesterol ester

Plasma and retinas were collected from RA, OIR and K604-treated OIR pups at P17. The levels of cholesterol esters were measured using luminescence-based Cholesterol/Cholesterol Ester Glo™ Assay (J3190, Promega, Madison, WI) as per manufacturers’ protocol. Briefly, retinas were homogenized, and blood plasma were diluted (1:10) in Cholesterol lysis solution and incubated at 37 °C for 30 min. Equal amounts of extracts were incubated in cholesterol detection reagent with and without cholesterol esterase enzyme in a 96 well white bottom plates at room temperature for 1 h. Luminescence was recorded using Polar Star Omega microplate reader (BMG Labtech Inc, NC). Total and free cholesterol concentrations were measured by comparing the luminescence of samples with and without cholesterol esterase, respectively. Cholesterol ester concentrations were calculated as the difference between total and free cholesterol concentrations.

### Detection of K604 in retina samples by LC–MRM–MS analysis (liquid chromatography–multiple reaction monitoring–mass spectrometry)

K604 is an small molecule capable of penetrating the blood–brain-barrier in experimental models [36]. To verify its ability to penetrate the blood–retinal-barrier, mice were treated with K604 (10 mg/kg) from P7 to P16 and retinas were collected for analysis on P17 (18 h after the last K604 injection). LC–MRM–MS analysis was performed to detect the K604 spectrum. Retina samples were processed as follows: 200 µL extraction buffer (acetonitrile:isopropanol:water = 4:4:2) was added to the retina sample tube together with 0.1 mL zirconium oxide bead (0.5 mm diameter, Next Advance). The sample tube was placed in a Bullet Blender (BBX24, Next Advance) and blended using Speed 8 for 3 min. Sample tubes were then centrifuged at 16,000×*g* for 10 min (room temperature) and the supernatant was transferred into a glass vial for LC-MRM-MS analysis.

Separation of extracted retina samples (5 µL) was performed using a Thermo Hypersil C8 column (50 × 2.1 mm, 1.9 µm) on a Shimadzu Nexera UHPLC system at a flowrate of 0.2 mL/min using a gradient elution from 5 to 95% acetonitrile (with 0.1% formic acid) in 6 min. The effluent was ionized using positive electrospray and analyzed on a TSQ Quantiva triple–quadrupole mass spectrometry with the following instrument settings: ion spray voltage 3500 V, sheath gas 35, ion transfer tube temperature 325, aux gas 10, and unit resolution for Q1/Q3. The optimal collision energy and RF lens setting were determined using purchased standards. The transitions monitored for K604 were as 503/353, 503/241, and 503/226.

The integrated peak areas for these transitions were calculated for each sample using Skyline software (version 20.0, University of Washington) and the most intense one (503/353) was used for quantification. The internal standard for K604 was performed at 1 pmol and the spectrum was detected at an intensity of 5530.124 equal to 1 pmol at 4.9 min. The K604 concentration for retinal samples detected at 4.9 min ranged from retina 0.3 pmol to 1.07 pmol (mean ± SEM = 0.66 ± 0.24, *n* = 3), indicating that the drug reaches the retina and persists over time.

### Statistical analysis

Differences among the groups were compared by an independent two sample *t* test for two groups or one-way ANOVA followed by a post hoc test for multiple comparisons. Values are represented as means ± standard error of the means (SEM). *P* values of less than 0.05 were considered significant.

## Results

### OIR-induced retinal neovascularization (RNV) involves upregulation of the LDL receptor (LDLR) and increases lipid accumulation and CE formation

**Fig. 1:**
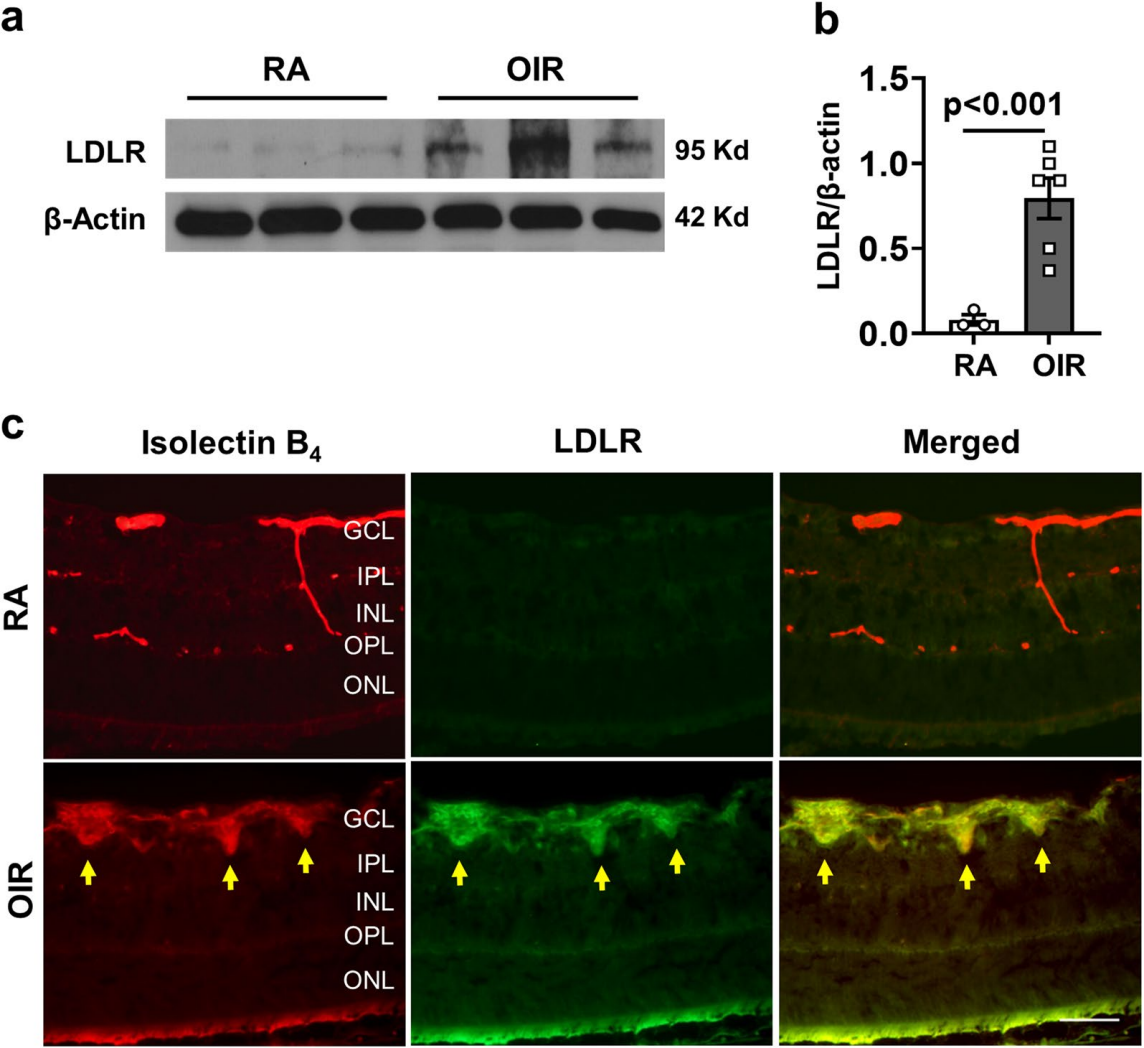
Expression of low-density lipoprotein receptor (LDLR) is increased during OIR-induced retinal neovascularization (RNV). **a**, **b** Western blotting and quantification show increased expression of LDLR in P17 OIR retinas, mean ± SEM, *n* = 3–6. **c** LDLR is colocalized with IB_4_-positive areas of RNV in OIR retina sections (yellow arrows) but is absent in room air (RA) control. *n* = 4, scale bar = 20 µm

We first examined the potential role of cholesterol metabolism in RNV by determining the effects of OIR on the expression and function of the LDL receptor (LDLR). Western blot analysis showed a marked increase in LDLR protein levels in the OIR retinas compared to the RA controls (Fig. 1a, b). Immunolabeling studies also showed prominent LDLR staining in the OIR retinas, whereas the RA controls were largely negative (Fig. 1c). LDLR immunoreactivity was strongly increased in the ganglion cell layer (GCL) of the OIR retina and was colocalized with IB_4_ positive areas of RNV. Weak immunoreactivity was also evident in the outer plexiform layer (OPL) and photoreceptor (PR) outer segments.

**Fig. 2:**
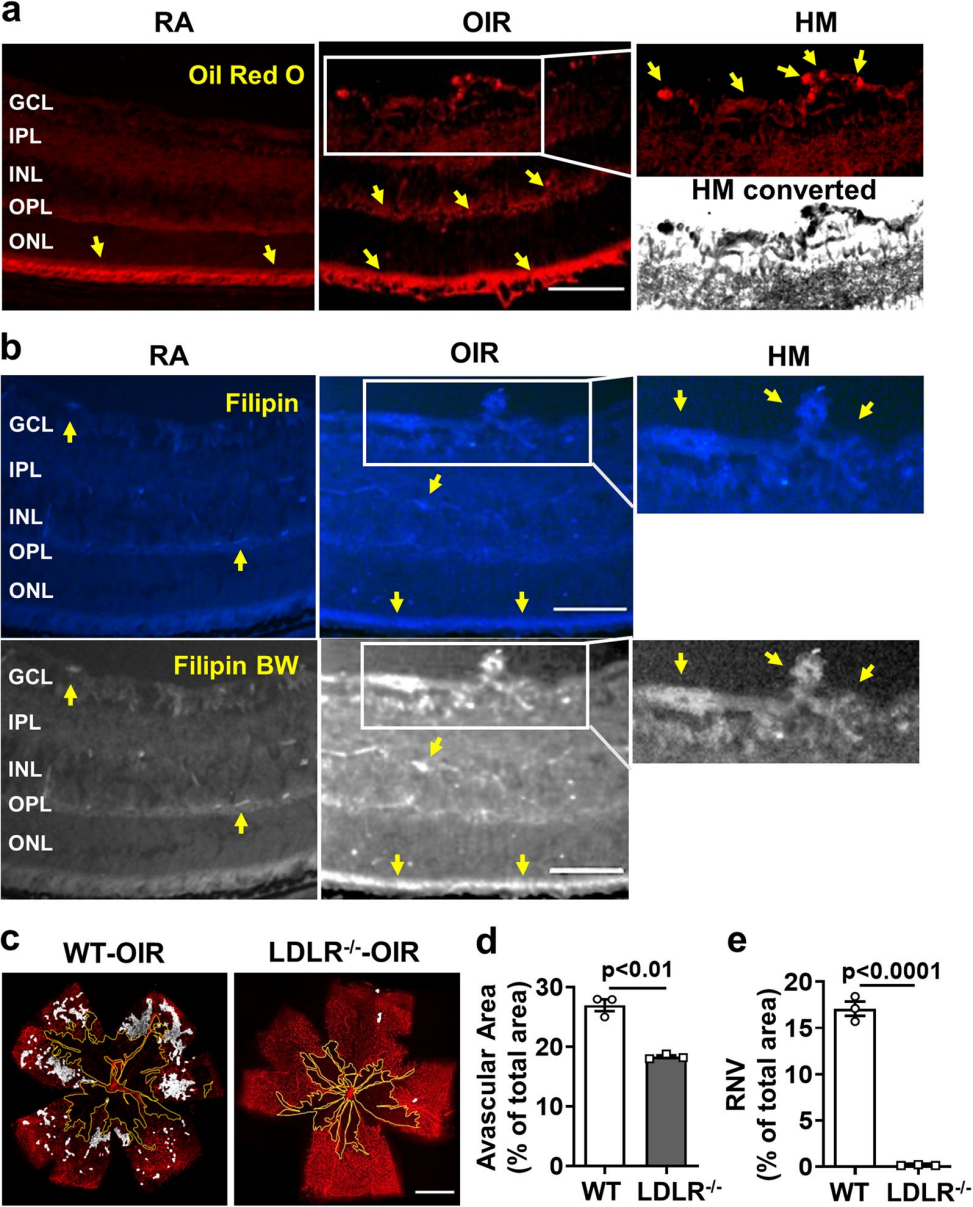
OIR-induced retinal neovascularization (RNV) is accompanied by lipid accumulation and blocked by LDLR deletion. **a** Oil red O staining of frozen sections shows intense reaction (yellow arrows) in areas of RNV in OIR retinas (indicated in high magnification (HM) image and HM converted to black and white). Intense Oil red O reaction is also evident in the outer plexiform layer (OPL) and photoreceptor (PR) outer segments of the OIR retina. Reaction is also evident in the outer segments of the RA control retina. *n* = 4, scale bar = 25 μm. **b** Detection of cholesterol ester (CE) by filipin staining of frozen retina sections shows prominent increases in CE formation in the ganglion cell, outer plexiform and photoreceptor layers of the OIR retina Filipin images are converted to black and white (BW) to show staining better. RA control sample shows a slight reaction to filipin in retinal vessel-like structures (arrows). *n* = 4, scale bar = 25 μm. **c**–**e** OIR-induced RNV (white highlighted areas) is almost completely blocked in LDLR^−/−^ mice and the avascular area (AVA, yellow outline) is significantly reduced. *n* = 3, scale bar = 300 µm

The areas of RNV of the OIR retina were also positive for Oil Red O and filipin staining (after extraction of unesterified cholesterol), indicating marked increased accumulation of cholesterol and other neutral lipids and deposition of CE, respectively (Fig. 2a, b). Oil Red O and filipin staining were also evident in inner retinal layers and outer segments of the OIR retina. RA control retinas showed weak Oil Red O and filipin reaction in the inner retinal layers and outer segments. We also examined the potential role of LDL uptake in the OIR-induced vascular injury by studies using LDLR knockout mice. Neonatal LDLR knockout mice, genetic control mice, and their nursing dams were maintained in hyperoxia from P7 to P12 and then returned to normoxia until P17 (Additional file 1: Fig. S1a). Analysis of IB_4_ labeled retinal flat mounts showed that deletion of LDLR almost completely blocked RNV and significantly reduced the avascular area (AVA) as compared with RA controls (Fig. 2c–e).

### OIR induces increased expression of ACAT1 and pro-inflammatory mediators

**Fig. 3:**
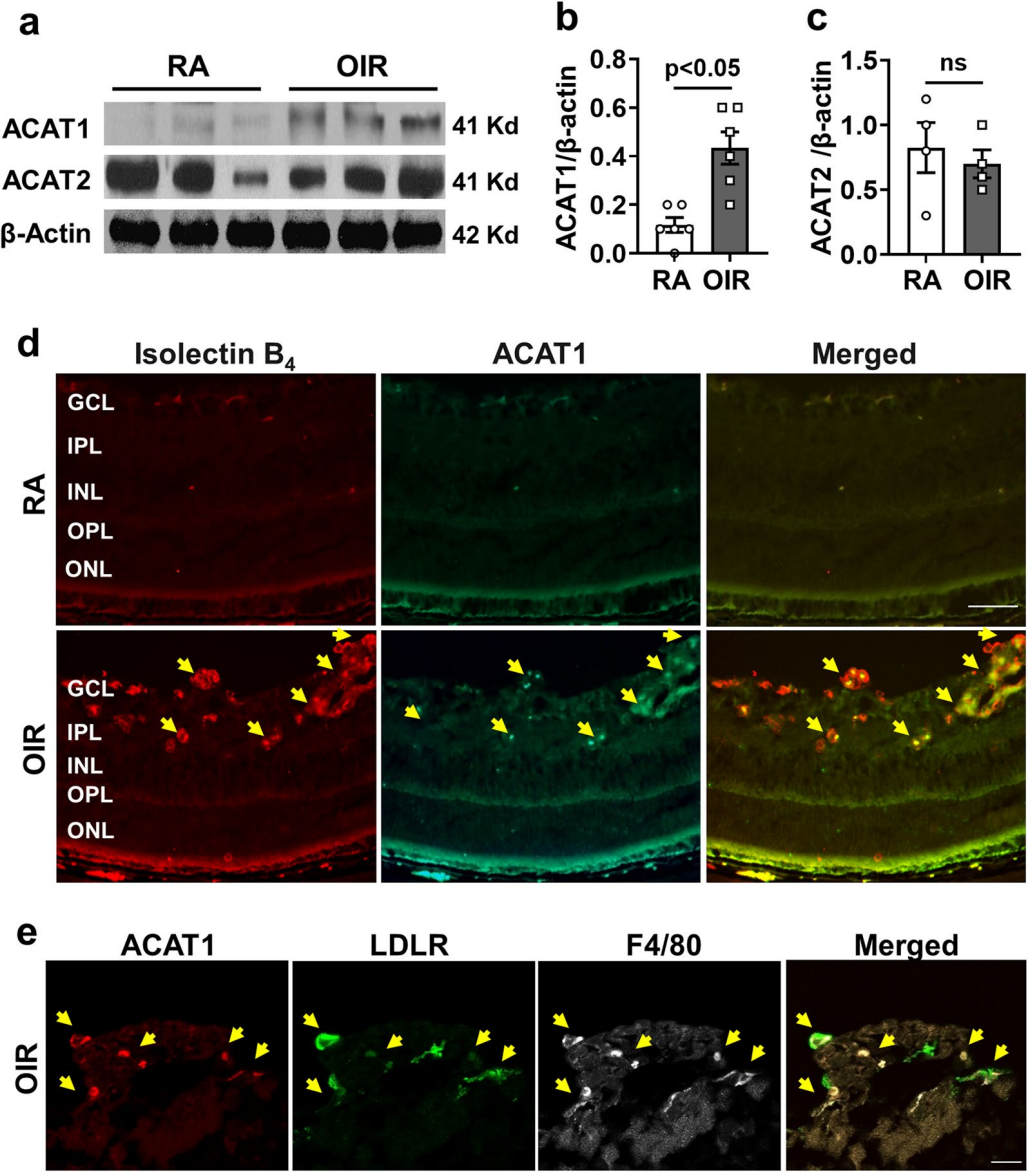
OIR induces increased expression of ACAT1. Mice were maintained in hyperoxia from P7 to P12 and returned to room air. Retina samples were collected at P17 and prepared for imaging and western blotting. **a** Representative immunoblot images and quantification showing upregulation of **b** ACAT1, and no change in **c** ACAT2 in the OIR retinas compared to RA controls. mean ± SEM, *n* = 4–6. **d** Representative retinal images show ACAT1 co-localized with IB_4_-positive retinal vessels in areas of RNV (yellow arrows). *n* = 4, scale bar = 60 μm. **e** High magnification images (×63) of an area of RNV tuft formation show ACAT1 co-localization with LDLR and F4/80 positive macrophage/microglial cells. *n* = 4, scale bar = 40 μm

The above results strongly support the role of cholesterol uptake in pathological RNV. However, targeting the LDLR is controversial as a potential therapy due to the risk of atherosclerosis and cardiovascular disease [37]. Studies in macrophages have shown that internalization of LDL cholesterol by the LDLR results in increases in intracellular cholesterol levels as well as increased ACAT1 activity leading to increases in CE formation [38]. We next examined ACAT1 protein levels and localization in the retina using Western blot and immunolabeling techniques. Western blot analysis of retina tissue extracts collected from OIR retinas at P17 confirmed significant increases in ACAT1 protein (Fig. 3a, b). Immunolocalization studies showed a marked increase in ACAT1 reactivity in the OIR retinas. The ACAT1 immunostaining was particularly prominent in IB_4_-positive areas of RNV but was also increased in the outer plexiform layer (OPL) and photoreceptor (PR) outer segments (Fig. 3d). Further analysis showed that ACAT1-positive cells within in the vitreoretinal neovascular tufts were also positive for the LDLR and the macrophage/microglia marker F4/80 (Fig. 3e). Western blot analysis of ACAT2 protein showed similar levels in the OIR and RA control retinas (Fig. 3a, c).

**Fig. 4:**
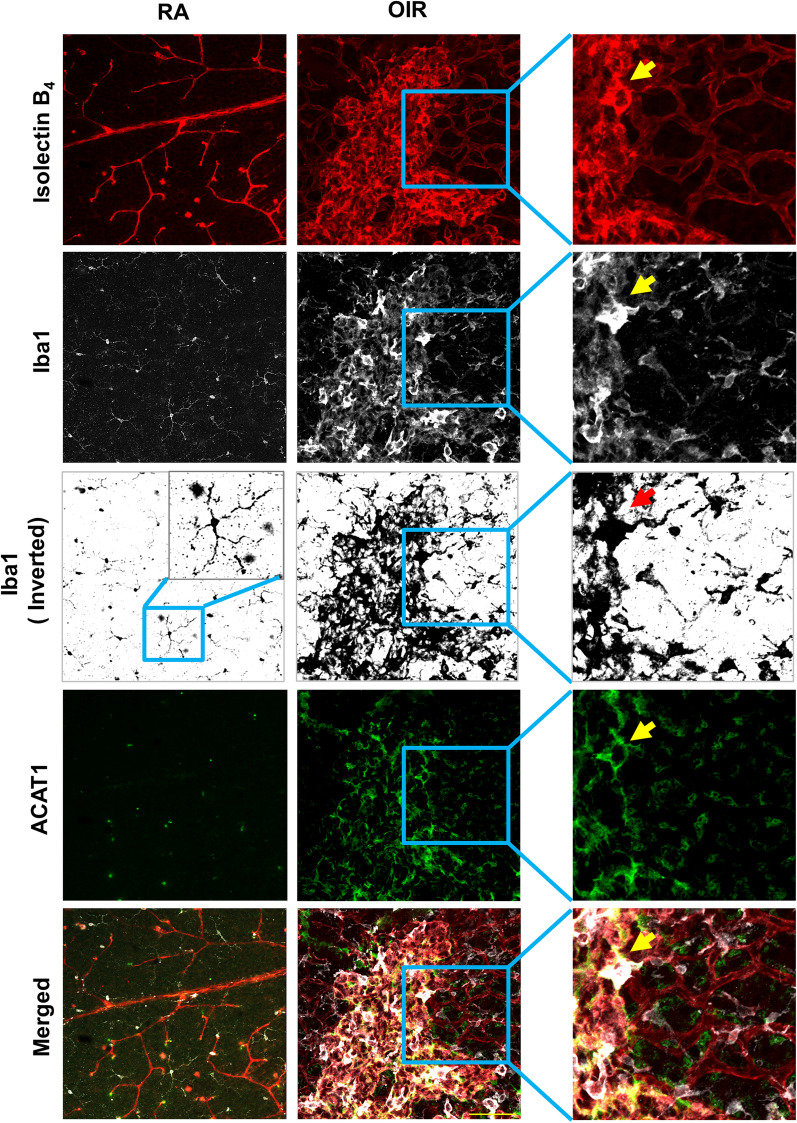
ACAT1 is colocalized with ameboid activated macrophage/microglia-like cells in areas of RNV. RNV area in the OIR retina shows colocalization of IB_4_ with the macrophage/microglia marker Iba1 and ACAT1. Insets show strong immunoreactivity for ACAT1 in ameboid shaped macrophage/microglia in the area of RNV of the OIR retina. ACAT1 expression is weak in Iba1-positive cells outside of the area of RNV of the OIR retina and ramified microglia of the RA control retina. *n* = 4, scale bar = 60 μm

**Fig. 5:**
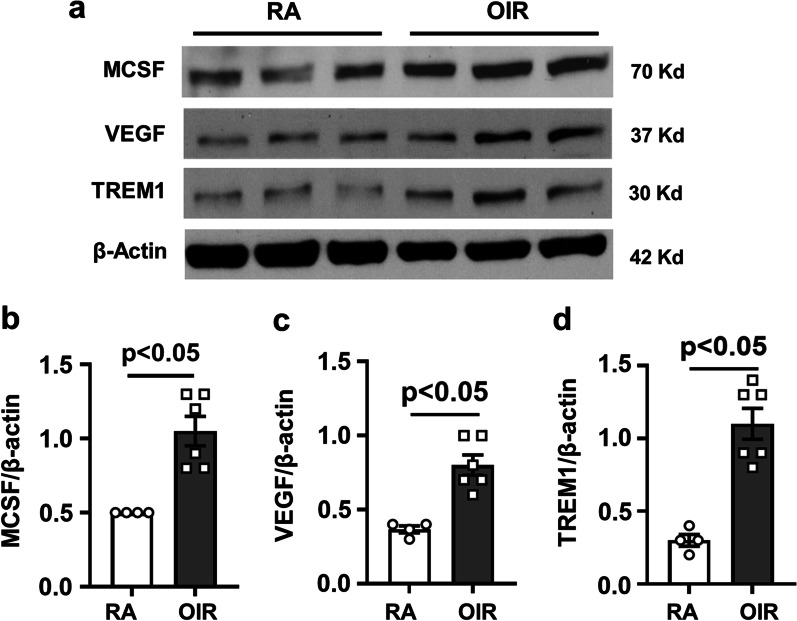
OIR induces increased expression of pro-inflammatory markers. Mice were maintained in hyperoxia from P7 to P12 and returned to room air. Retina samples were collected at P17 and prepared for imaging and western blotting. **a** Representative immunoblot images and **b**–**e** quantification show upregulation of MCSF (**a**, **b**), VEGF (**a**, **c**), and TREM1 (**a**, **d**) in the OIR retinas compared to RA controls. mean ± SEM, *n* = 4–6

Previous studies in the mouse model of OIR have shown that activation of macrophage/microglia has a critical role in RNV [39]. Immunolabeling of retina flat mounts showed colocalization of ACAT1 with IB_4_, and Iba1 in areas of RNV of OIR mice at P17 (Fig. 4). The ACAT1 immunoreactivity was concentrated in the areas of RNV and was strongly expressed in cells with an ameboid profile. Ramified microglia in RA control retinas and the normally vascularized zones adjacent to the RNV areas were weakly positive for ACAT1. Immunolabeling analysis of retinal sections showed that ACAT1 expression was colocalized in cells positive for macrophage/microglial cell markers F4/80 and Iba1, as well as with TREM1- and MCSF-positive cells in the inner retina/vitreous interface in the OIR retina (Additional file 2: Fig. S2a–d). ACAT1 expression was also co-localized with TREM1 in the OPL and photoreceptor/RPE interface (Additional file 2: Fig. S2c) and with MCSF-positive vessel-like structures in the OPL (Additional file 2: Fig. S2d). Western blot analysis of retina tissue extracts collected from OIR retinas at P17 confirmed significant increases in MCSF, VEGF, and TREM1 as compared with the room air (RA) control (Fig. 5a–d).

### Inhibiting ACAT limits OIR-induced increases in RNV

Next, we evaluated the potential role of ACAT in OIR-induced RNV by treating OIR mouse pups with an ACAT inhibitor (*N*-[3-(4-hydroxyphenyl)-1-oxo-2-propenyl]-l-phenylalanine, methyl ester) that inhibits both ACAT isoforms [26]. Retinal flat mounts labeled with IB_4_ showed an extensive area of RNV (~ 19.1% of total area) and a large AVA (~ 16.4% of the total area) in the vehicle-treated group. The ACAT1/ACAT2 inhibitor treatment significantly reduced RNV and AVA (Additional file 3: Fig. S3a–c).

**Fig. 6:**
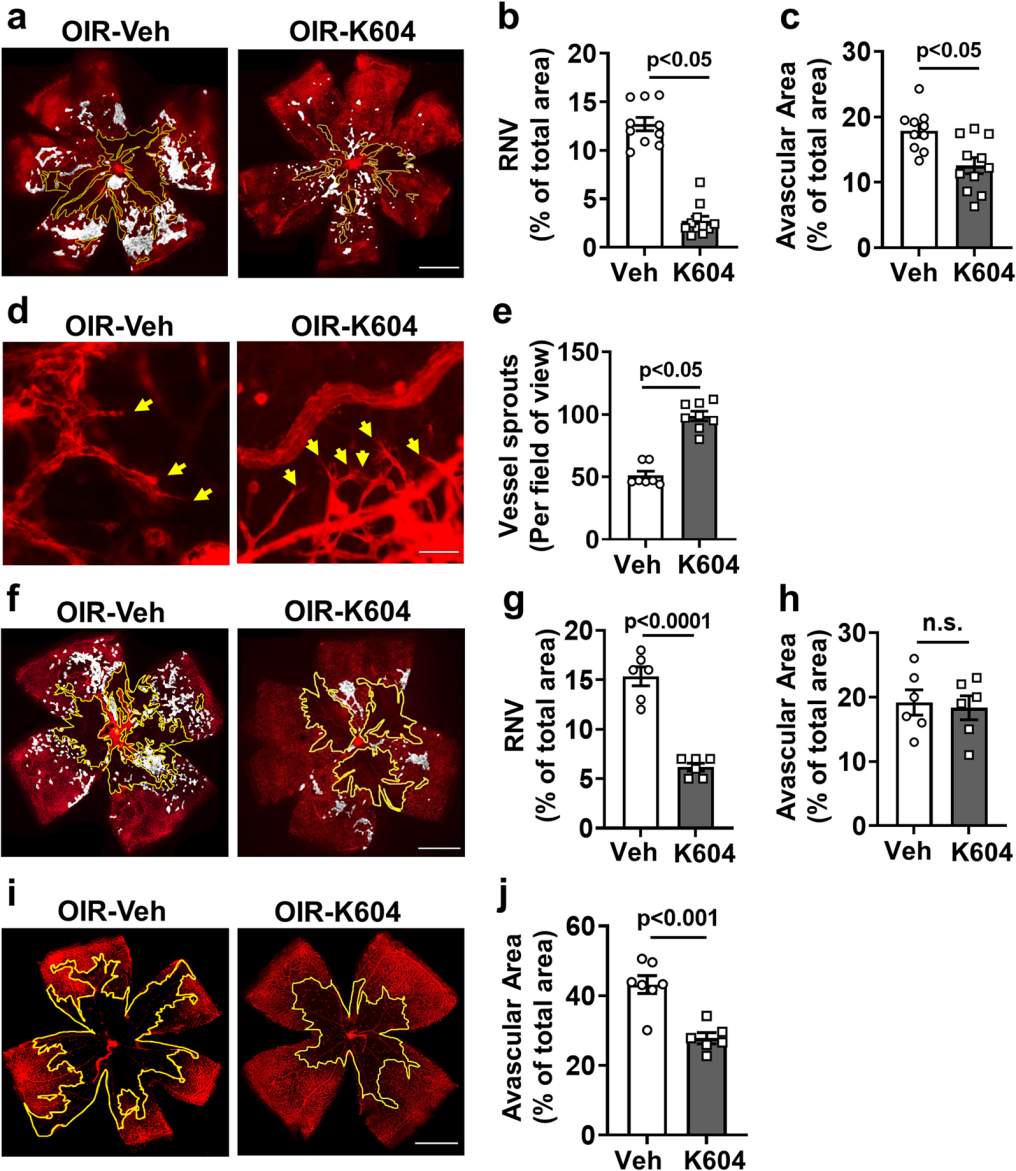
Inhibiting ACAT1 decreases retinal neovascularization (RNV) and avascular area (AVA) during OIR. **a**–**e** OIR mice were treated with the ACAT1 inhibitor K604 or vehicle from P7 to P16. Eyes were enucleated at P17 and retinal vessels were visualized by IB_4_ labeling of retinal flat mounts. **a**–**c** Areas of RNV (white highlighted areas) and AVA (yellow outline) were significantly reduced by treatment with K604. *n* = 10–11, scale bar = 300 μm. **d**, **e** K604 treatment increased vascular sprouting as shown by increased numbers of tip cells with filopodia, as compared to vehicle treated OIR retinas. *n* = 7–8, scale bar = 40 µm. **f**–**h** OIR mice were treated with the ACAT1 inhibitor K604 or vehicle from P12 to P16. Eyes were enucleated at P17 and retinal vessels were visualized by IB_4_ labeling. Treatment with K604 significantly reduced the formation of RNV (white highlighted areas), but not the AVA (yellow outline). *n* = 10–11, scale bar = 300 μm. **i**, **j** OIR mice were treated with the ACAT1 inhibitor K604 or vehicle from P7 to P11. Eyes were enucleated at P12 and retinal vessels were visualized by IB_4_ labeling**.** Treatment with K604 significantly reduced the AVA (yellow outline). *n* = 6–7, scale bar = 300 μm

Previous studies have shown that ACAT1 is the predominant isoform in the retina [9]. Thus, we next determined the effects of the specific ACAT1 inhibitor K604 on OIR-induced RNV. K604 has been shown to be effective as a therapy in other diseases, including Alzheimer’s and cancer [17, 18]. Neonatal mice were treated with K604 from P7 to P16, and retinas were collected on P17. The imaging analysis of retinal flat mounts showed that the K604 treatment significantly prevented RNV and reduced the AVA compared to the vehicle group (Fig. 6a–c). In contrast with these K604-induced decreases in RNV in the K604-treated OIR retinas, vascular sprouting into the AVA was significantly increased as shown by quantification of IB_4_-positive endothelial tip cells at the boundary of the AVA (Fig. 6d, e). Tip cells are specialized extensions of endothelial cells from preexisting vessels and are required for physiological angiogenesis. They have been shown to play a key role in retinal vascular development and repair [40–42]. These data suggest that K604 treatment enhances vascular repair as well as inhibiting pathological RNV (Fig. 6d, e). However, treatment with K604 during the hypoxia phase of OIR reduced the RNV but did not alter the AVA (Fig. 6f–h), whereas K604 treatment during the hyperoxia phase of OIR significantly reduced the AVA (Fig. 6i, j). Taken together, these data suggest a protective action of K604 in limiting the hyperoxia-induced vaso-obliteration while allowing physiological vascular sprouting, and inhibiting pathological RNV.

### Specific inhibition of ACAT1 with K604 decreases lipid accumulation and CE formation in P17 OIR retinas

**Fig. 7:**
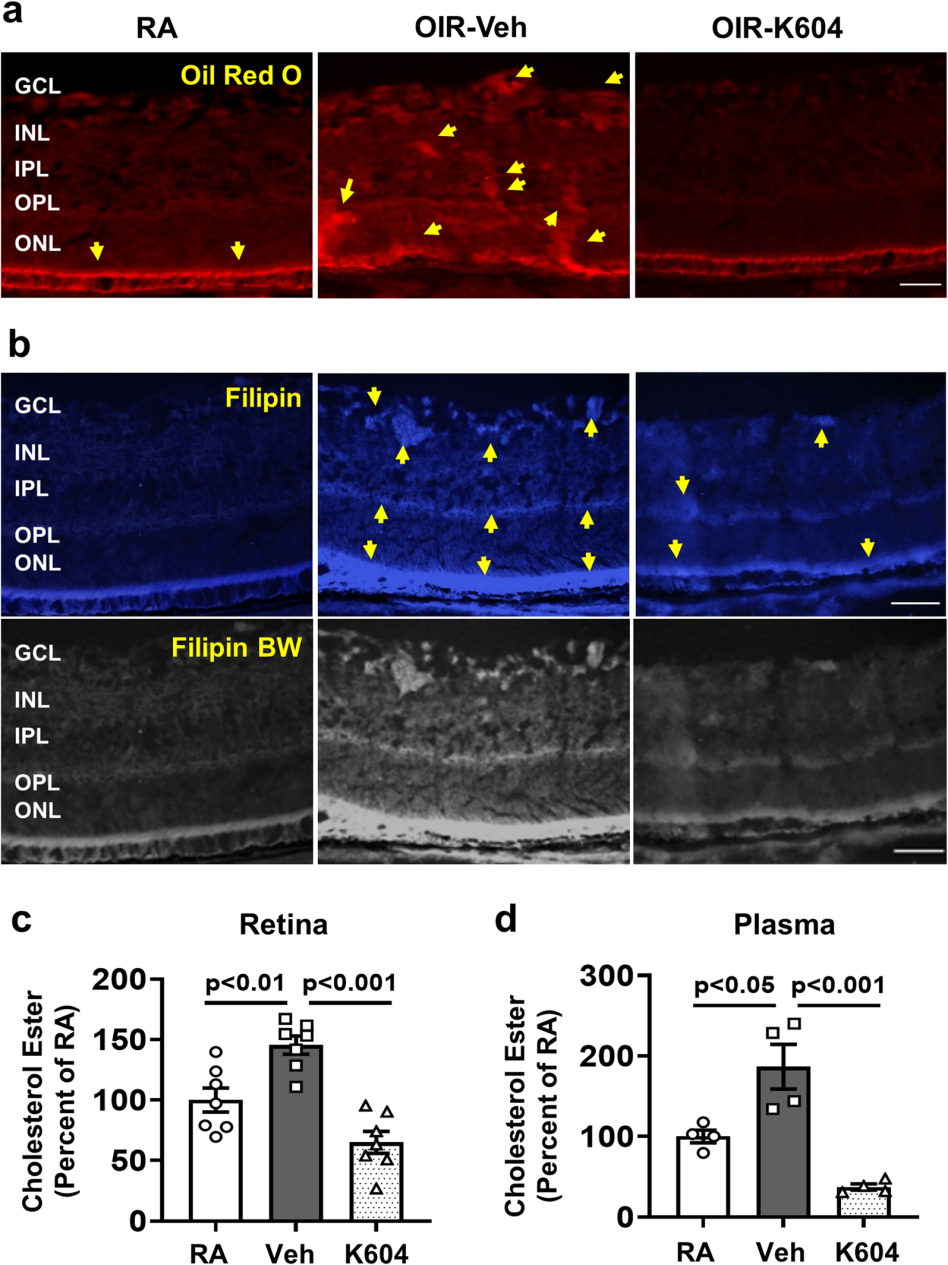
ACAT1 inhibition suppresses lipid uptake and CE formation in OIR retinas and plasma. OIR mice were treated with K604 or vehicle from P7 to P16. **a**, **b** Frozen sections from vehicle treated OIR retinas show increased reactivity (yellow arrows) for neutral lipids and cholesterol ester (CE) as detected by Oil Red O (**a**) and filipin staining (**b**), compared with the RA control retinas. Filipin images are converted to black and white (BW) to show the staining better. Treatment with K604 prevented the lipid accumulation and CE formation in the OIR retina. *n* = 3, scale bar = 20–25 μm. Cholesterol levels were measured in retinas and plasma from RA, OIR and K604-treated OIR pups at P17. **c**, **d** Biochemical analysis of cholesterol levels in retina and plasma samples showed upregulation of cholesterol ester in the vehicle treated OIR animals. K604 treatment completely blocked this effect. mean ± SEM, *n* = 4–7

Imaging studies using Oil Red O and filipin labeling showed that the K604 treatment also normalized the patterns of neutral lipid and cholesterol ester distribution in the OIR retina, respectively, as compared to the vehicle treated OIR samples (Fig. 7a, b). Biochemical analyses of retina and plasma samples showed increased levels of cholesterol ester in samples from the vehicle treated OIR mice as compared with the RA controls. K604 treatment completely blocked this effect (Fig. 7c, d). Consistent with the OIR-induced increases in lipid uptake and CE formation and their suppression by K604 treatment, immunolocalization studies showed strong immunoreactivity for LDLR and ACAT1 in F4/80-positive cells of the OIR vehicle control retina (Additional file 4: Fig. S4). These profiles were absent in the retinas of the K604 treated OIR mice.

### Specific inhibition of ACAT1 with K604 decreases expression of ACAT1 and pro-inflammatory mediators in P17 OIR retinas

**Fig. 8:**
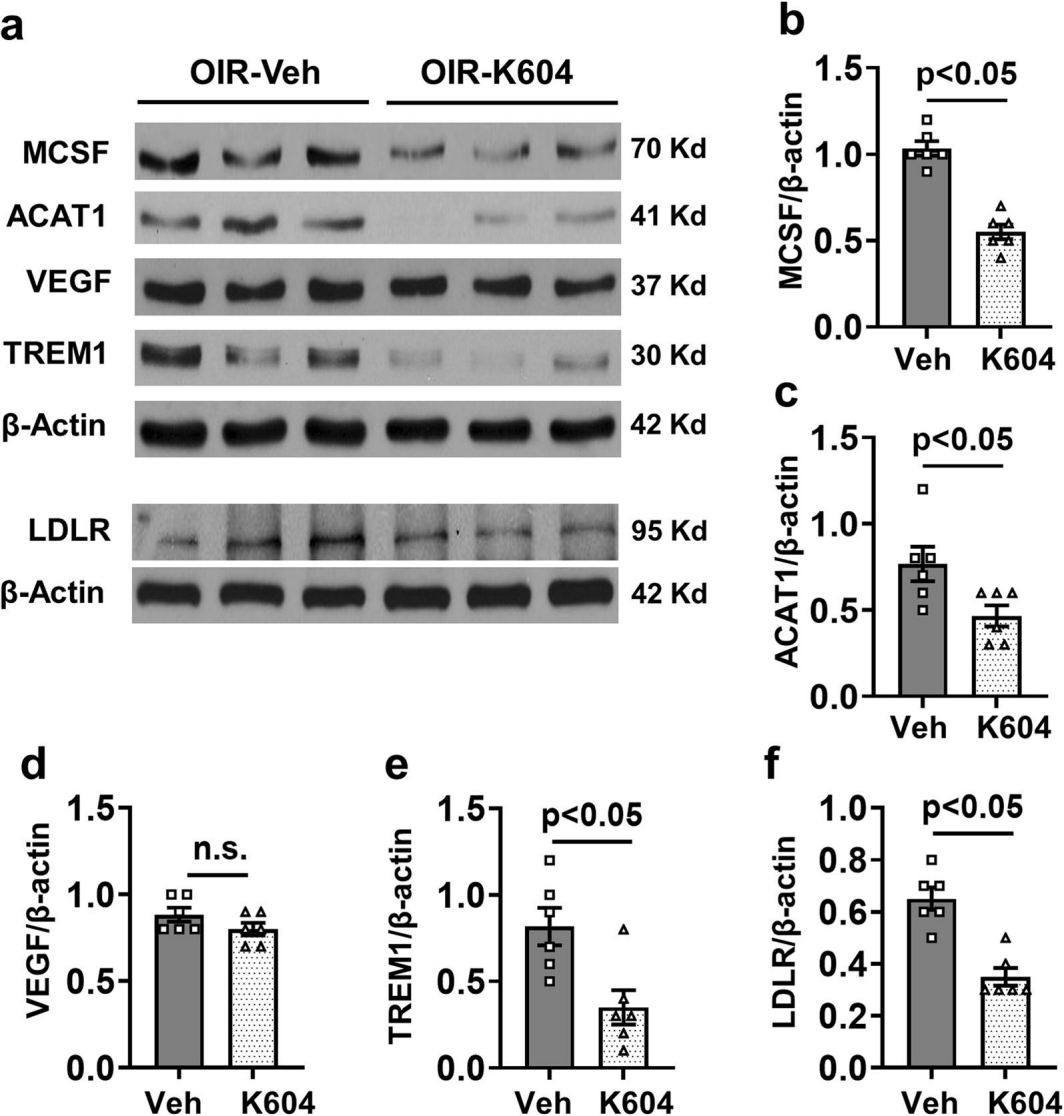
ACAT1 inhibition decreases expression of ACAT1 and inflammatory mediators. OIR mice were treated with K604 or vehicle from P7 to P16. Retinal lysates were prepared at P17 for Western blotting. **a** Representative western blots and **b**–**f** their quantification shows that ACAT1 inhibition significantly suppresses expression of MCSF (**a**, **b**), ACAT1 (**a**, **c**), TREM1 (**a**, **e**), and LDLR (**a**, **f**), as compared with the vehicle control. However, K604 treatment did not alter levels of VEGF (**a**, **d**). mean ± SEM, *n* = 6

Further studies examined the effects of inhibiting ACAT1 with K604 on the expression of ACAT1 and pro-inflammatory mediators in the OIR retina. Western blot analysis showed that the K604 treatment significantly suppressed the expression of ACAT1, MCSF, TREM1, and LDLR in the OIR retina compared to the vehicle-treated controls (Fig. 8a–c, e, and f). However, levels of VEGF were not altered by the K604 treatment (Fig. 8a, d).

**Fig. 9:**
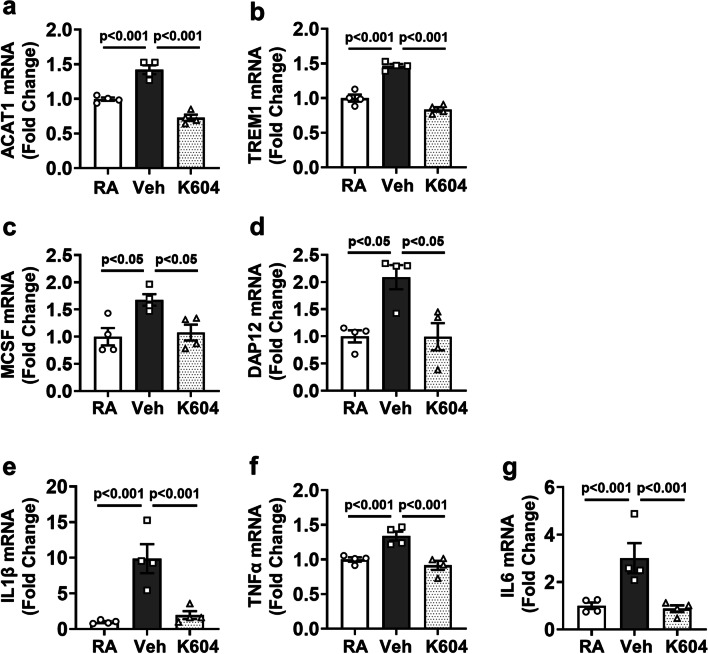
Inhibiting ACAT1 reduces mRNA for ACAT1 and associated inflammatory mediators in OIR retinas. OIR mice were treated with the ACAT1 inhibitor K604 or vehicle from P7 to P16. Retinas were enucleated at P17 and gene expression was analyzed by RT-PCR. **a**–**g** Analysis of mRNA levels shows upregulation of **a** ACAT1, **b** TREM1, **c** MCSF, and **d** DAP12, along with the inflammatory markers **e** IL1β, **f** TNFα, and **g** IL6 in OIR retinas, compared to RA control. K604 treatment significantly reduced their expression as compared to vehicle treated OIR-retinas. mean ± SEM, *n* = 4

Quantitative RT PCR analysis confirmed significant increases in mRNA levels for ACAT1, TREM1, DAP12, MCSF, TNFα, IL6, and IL1β in retinas of the OIR vehicle-treated group as compared to the room air controls. Each of these increases was blocked by the K604 treatment (Fig. 9).

### K604 treatment attenuates hypoxia-induced expression of ACAT1 and pro-inflammatory mediators in human microglia and macrophage cells

**Fig. 10:**
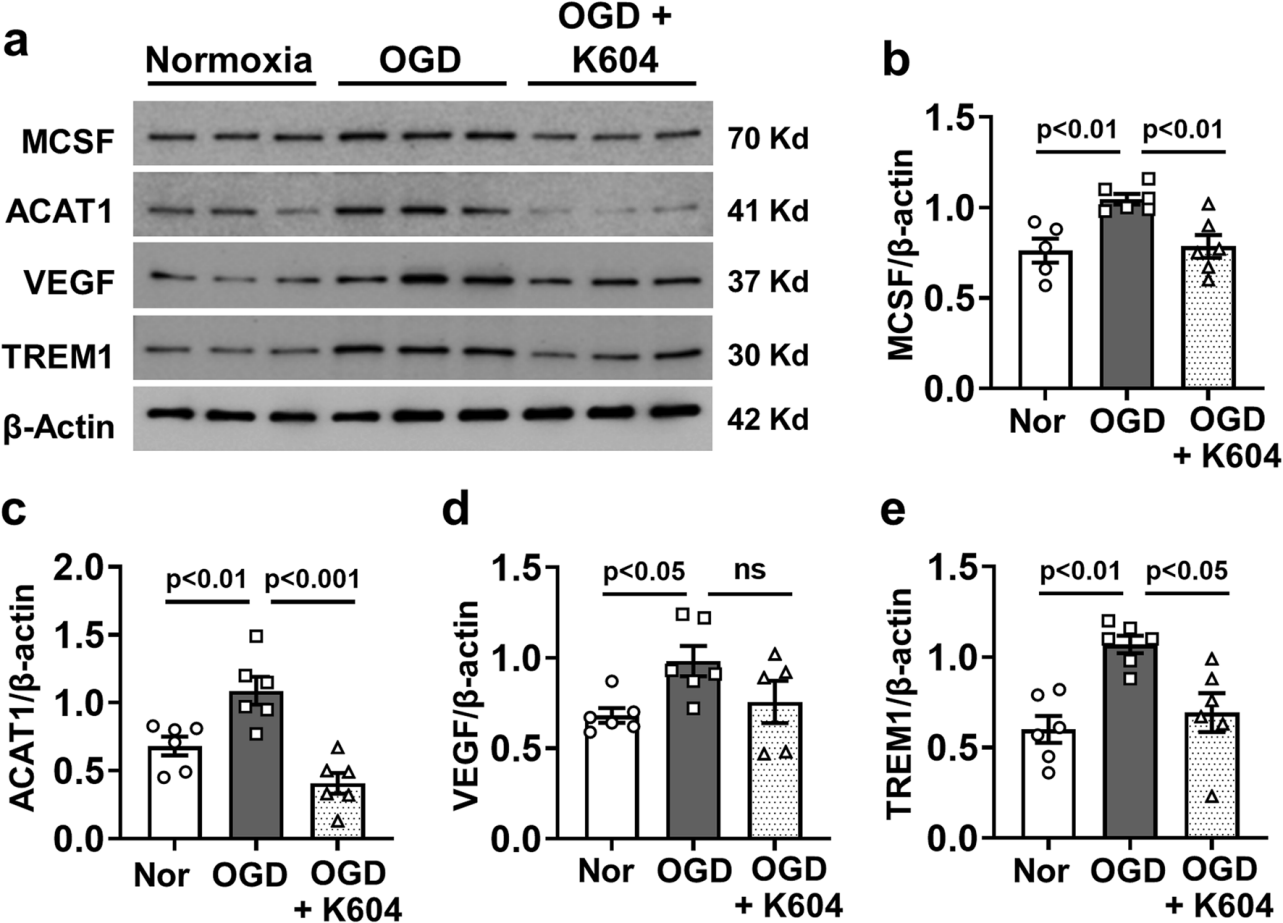
Inhibiting ACAT1 attenuates OGD-induced increases in expression of ACAT1 and inflammatory mediators in HMC3 microglia. Human microglial cells were subjected to OGD (1% O_2,_ glucose-free medium) or normal media (21% O_2,_ 25 mM glucose) with K604 or vehicle for 24 h. **a** Western blotting, and **b**–**e** quantification shows upregulation of MCSF (**a**, **b**), ACAT1 (**a**, **c**), and TREM1 (**a**, **e**) after hypoxia treatment as compared with normoxia. K604 treatment markedly suppressed all except VEGF (**a**, **d**). mean ± SEM, *n* = 6

To examine the role of ACAT1 activity in microglial cell expression of inflammatory mediators under ischemic/hypoxic conditions, we performed in vitro studies using human HMC3 microglia exposed to oxygen and glucose deprivation (OGD). This treatment significantly increased the levels of ACAT1, MCSF, and TREM1 compared with normoxia control cells. Those increases were blocked by treatment with K604 (1 µM) (Fig. 10a–c, and e). However, the expression of VEGF was not significantly changed by OGD or K604 treatment (Fig. 10a, d). Studies using THP1 macrophages showed a similar effect of hypoxia treatment in low glucose media (Additional file 5: Fig. S5).

## Discussion

This study demonstrates for the first time that activation of ACAT1 and CE formation promote pathological RNV in the mouse model of OIR by a mechanism associated with upregulation of TREM1, MCSF, and other inflammatory mediators. Previously we have shown that TREM1 is strongly implicated in the pathogenesis of RNV during OIR. We found that specific inhibition of TREM1 significantly blocked pathological RNV and decreased the AVA along with significantly reducing the expression of TREM1, MCSF, and VEGF [25]. TREM1 is a receptor of the immunoglobulin superfamily. It amplifies inflammatory signaling through activation of its internal adaptor DAP12. DAP12 is a multifunctional transcription factor that amplifies expression of IL6, IL1β, TNFα, among others [43].

Here we show that the hypoxia phase of OIR induces increased expression of LDLR along with increases in lipid accumulation, upregulation of ACAT1 expression, and elevated CE formation in areas of RNV (Figs. 1, 2, 3 and 4). To assess the involvement of LDLR-mediated cholesterol uptake in the pathogenesis of OIR-induced RNV, we used mice with global deletion of LDLR. Our data showed that deletion of the LDLR gene almost completely blocked the formation of RNV in the OIR mice and significantly reduced the AVA (Fig. 2c–e). The potential role of LDLR and cholesterol metabolism in OIR-induced RNV is further evidenced by our data showing prominent immunoreactivity for LDLR and ACAT1 in areas of vitreoretinal neovascularization (Fig. 3e, Additional file 4: Fig. S4). This finding underscores the significant role of cholesterol metabolism in the OIR-induced vascular injury. Moreover, the co-localization of LDLR and ACAT1 in F4/80-positive macrophage/microglia (Fig. 3e, Additional file 4: Fig. S4) is consistent with the well-established role of these cells in the pathology [31, 39].

A biochemical assay confirmed significant increases in CE levels in retinal samples at the time of peak RNV formation during the hypoxia phase of OIR (Fig. 7c). These increases in lipid uptake and CE formation were accompanied by significant increases in protein levels of LDLR, ACAT1, TREM1, MCSF, VEGF (Figs. 1, 3, and 5). All alterations except for the increase in VEGF were blocked by treatment with a specific inhibitor of ACAT1, K604 (Fig. 8). Interestingly, the cholesterol ester levels in retinas of the K604 treated OIR mice were slightly reduced as compared with the RA controls and there was a trend toward reduction in the plasma samples (Fig. 7d). These decreases are likely due to the systemic effects of the K604 treatment. However, it should be noted that K604 is known to reduce ACAT1 activity without completely blocking it [9].

Quantitative RTPCR analysis confirmed significant increases in mRNA for ACAT1, TREM1, and MCSF during the hypoxia phase of OIR (Fig. 9). These increases were accompanied by increases in mRNA for DAP12, IL6, IL1β, and TNFα, underscoring the OIR-induced amplification of inflammatory signaling. Each of these increases was blocked by treatment with the ACAT1 inhibitor, implying the specific role of ACAT1 activity in promoting this inflammatory process.

We further demonstrated the involvement of ACAT1 activity in the OIR-induced retinal vascular injury by studies showing that specific inhibition of ACAT1 significantly reduced RNV and reduced the avascular area in the OIR retina (Fig. 6). Our data showed that treatment with the ACAT1 inhibitor limits pathological RNV and increases vascular sprouting and that treatment during the hyperoxia phase reduces the vaso-obliteration.

The above findings were supported by immunolocalization studies showing high levels of ACAT1 immunoreactivity in IB_4_-positive areas of RNV (Fig. 4). The ACAT1-positive cells were also positive for the inflammatory mediators TREM1 and MCSF and the macrophage/microglial markers, F4/80 and Iba1 (Additional file 2: Fig. S2). These results indicate that hypoxia induces increased expression of ACAT1, TREM1, and MCSF in areas of RNV and macrophage/microglial cell activation.

Further study is required to evaluate the specific role of ACAT1 in the activation of macrophage/microglial cells during OIR-induced vascular injury and pathological RNV. However, our experiments using human microglia showed that oxygen/glucose deprivation caused significant increases in their expression of ACAT1, TREM1, and MCSF and that treatment with K604 completely blocked these increases (Fig. 10). Our future studies using cell-specific ACAT1 gene deletion will examine the relative contributions of myeloid-derived cells versus microglia and resident macrophages to the pathology.

LDL cholesterol is incorporated in cells through increased expression of the LDLR [44]. Studies in other fields have shown that macrophages ingest cholesterol by endocytosis of aggregated and native LDL via LDLR [45]. Under physiological conditions the lipid-scavenging function of macrophages is beneficial, but under continued hypoxic conditions, increased lipid uptake leads to excessive formation of CE, which results in foam cell formation [46].

Our results indicate that activity of ACAT1 is a key factor in the pathogenesis of retinal damage during ischemic retinopathy. Although our focus is on pathological RNV, specific inhibition of ACAT1 showed another beneficial effect in limiting the expansion of the avascular area. Here, we have demonstrated that increased levels of CE formation and upregulated expression of ACAT1, TREM1, MCSF, VEGF, and LDLR are associated with the OIR-induced expansion of the avascular area and development of RNV following hyperoxia treatment. Furthermore, all these changes except for the increase in VEGF could be significantly prevented by the systemic administration of a specific inhibitor of ACAT1, K604.

K604 is a small molecule that can penetrate blood brain barrier [36] and our LC–MRM–MS analysis results showed significant concentrations of K604 in retinal samples collected at 16 h after the last i.p. injection. This data indicates that the drug reaches the retina after systemic injection and persists over time and suggests that K604 is suitable for use in treatment of retinopathies characterized by pathological RNV.

Importantly, the ACAT1 inhibitor treatment did not affect levels of VEGF (Fig. 8d), indicating that specific inhibition of ACAT1 prevents RNV and improves vascular repair independently of an effect on VEGF expression. This is significant for the clinical development of this therapy. In premature infants, the retina and other organs are still in the process of development in which VEGF plays a primordial role [47]. Thus, anti-VEGF therapy could have adverse effects on development of the retina or other immature tissues. In addition, intravitreal injections of anti-VEGF or VEGF inhibitors are not effective for some patients [4]. Our data showed that a treatment with a specific ACAT1 helped to promote repair of the avascular area by promoting tip cell sprouting (Fig. 6d, e). VEGF has been shown to guide angiogenic sprouting by endothelial tip cell filopodia [41].

## Conclusion

In this study we have identified a novel mechanism of RNV that involves a link between the cholesterol pathway (LDLR, ACAT1, CE) and activation of the inflammatory mediators TREM1 and MCSF. Hypoxia increases LDL cholesterol uptake into retinal cells leading to activation of ACAT1. This, in turn, causes the accumulation of toxic levels of CE in retinas and promotes retinal inflammation characterized by upregulation of TREM1, MCSF, and inflammatory cytokines. Systemic inhibition of ACAT1 could represent a new target to treat pathological RNV in ischemic retinopathies without altering levels of VEGF and avoiding the side effects of intravitreal injections. Blocking the formation of CE in ischemic retinas offers a new therapeutic option to treat those retinopathies, where toxic levels of lipids are present. This study will be extended to investigate the specific participation of myeloid derived cells versus microglia in this model using mice with cell-specific deletion of the ACAT1 gene in myeloid-derived cells versus microglial cells.

## Supplementary Information

**Additional file 1: Figure S1.** Schematic representation of experimental groups, treatments, and timepoints for analyses. **a** WT and LDLR^−/−^ pups were subjected to OIR at P7 and sacrificed on P17. **b**–**d** WT pups were treated with ACAT inhibitor or vehicle from **b** P7 to P16 and sacrificed on P17 or **c** P12 to P16 and sacrificed on P17. **d** WT pups were treated with ACAT inhibitor or vehicle from P7 to P11 and sacrificed on P12.

**Additional file 2: Figure S2.** ACAT1 expression is colocalized with F4/80, Iba1, TREM1, and MCSF in OIR retinas. Mice were maintained in hyperoxia from P7 to P12 and then returned to room air. Eyes were enucleated at P17 and frozen sections were prepared for immunofluorescence imaging. ACAT1 is colocalized with **a** F4/80, **b** Iba1, **c** TREM1, **d** MCSF in areas of RNV. The areas of colocalization are indicated by yellow arrows. *n* = 4–6, scale bar = 60 μm.

**Additional file 3: Figure S3.** Inhibition of ACAT1/ACAT2 decreases RNV and AVA area in OIR retinas. OIR mice were treated with the ACAT1/ACAT2 inhibitor or vehicle from P7 to P16. Eyes were enucleated at P17 and prepared for retina flatmount analysis. **a** Retinal vessels were visualized by IB_4_ labeling and RNV (white highlighted areas) and AVA (yellow outline) were quantified. **b**, **c** Treatment with ACAT1/ACAT2 inhibitor significantly reduced the formation of RNV and decreased the AVA. *n* = 5–9, scale bar = 300 μm.

**Additional file 4: Figure S4.** ACAT1 inhibition suppresses OIR-induced increase in ACAT1, LDLR, and F4/80-positive perivascular macrophage/microglia. OIR mice were treated with K604 or vehicle from P7 to P16 and frozen sections were prepared for immunofluorescence imaging. K604 treatment suppressed the OIR-induced increase in ACAT1, LDLR, and F4/80-positive perivascular macrophage/microglia. *n* = 4, scale bar = 40 μm.

**Additional file 5: Figure S5.** Inhibiting ACAT1 attenuates hypoxia-induced increases in expression of ACAT1 and inflammatory mediators in THP1 macrophages. Human THP1 cells were cultured in RPMI medium containing 5 mM glucose and 2% FBS and subjected to hypoxia (1% O_2_) or normoxia (21% O_2_) with K604 or vehicle for 16 h. **a** Western blotting, and **b**–**e** quantification shows upregulation of MCSF (**a**, **b**), ACAT1 (**a**, **c**), and TREM1 (**a**, **e**) after hypoxia treatment as compared with normoxia. K604 treatment markedly suppressed all except VEGF (**a**, **d**). Mean ± SEM, *n* = 6.

**Additional file 6: Figure S6.** Images of no primary antibody controls.

**Additional file 7: Figure S7.** Uncropped images of Western blots.

**Additional file 8: Figure S8.** Uncropped images of Western blots.

**Additional file 9: Table S1.** List of reagents used in this study.

**Additional file 10: Table S2.** Primer sequences used in qRT-PCR.

## Data Availability

The data generated and/or analyzed during the current study are available from the corresponding author on reasonable request.

## Abbreviations

ACAT: Acyl-coenzyme A cholesterol transferase
AVA: Avascular area
BSA: Bovine serum albumin
CE: Cholesterol ester
DAP12: DNAX-activating protein 12
DR: Diabetic retinopathy
Iba1: Ionized calcium-binding adaptor molecule 1
IB_4_: Isolectin B_4_
IL1β: Interleukin 1β
IL6: Interleukin 6
i.p: Intraperitoneal
LDL: Low-density lipoprotein
LDLR: LDL receptor
MCSF: Macrophage colony stimulating factor
OIR: Oxygen-induced retinopathy
PBS: Phosphate buffer saline
P17: Postnatal day 17
qRT PCR: Quantitative real time PCR
RA: Room air
RNV: Retina neovascularization
ROP: Retinopathy of prematurity
TNFα: Tumor necrosis factor α
TREM1: Triggering receptor expressed on myeloid cells 1
UC: Unesterified cholesterol
VEGF: Vascular endothelial growth factor
