# Supplementary figures and images for: Role of acyl-coenzyme A: cholesterol transferase 1 (ACAT1) in retinal neovascularization
Source: J Neuroinflammation. 2023 Jan 23;20:14. doi: 10.1186/s12974-023-02700-5 (PMC9869542; doi:10.1186/s12974-023-02700-5)

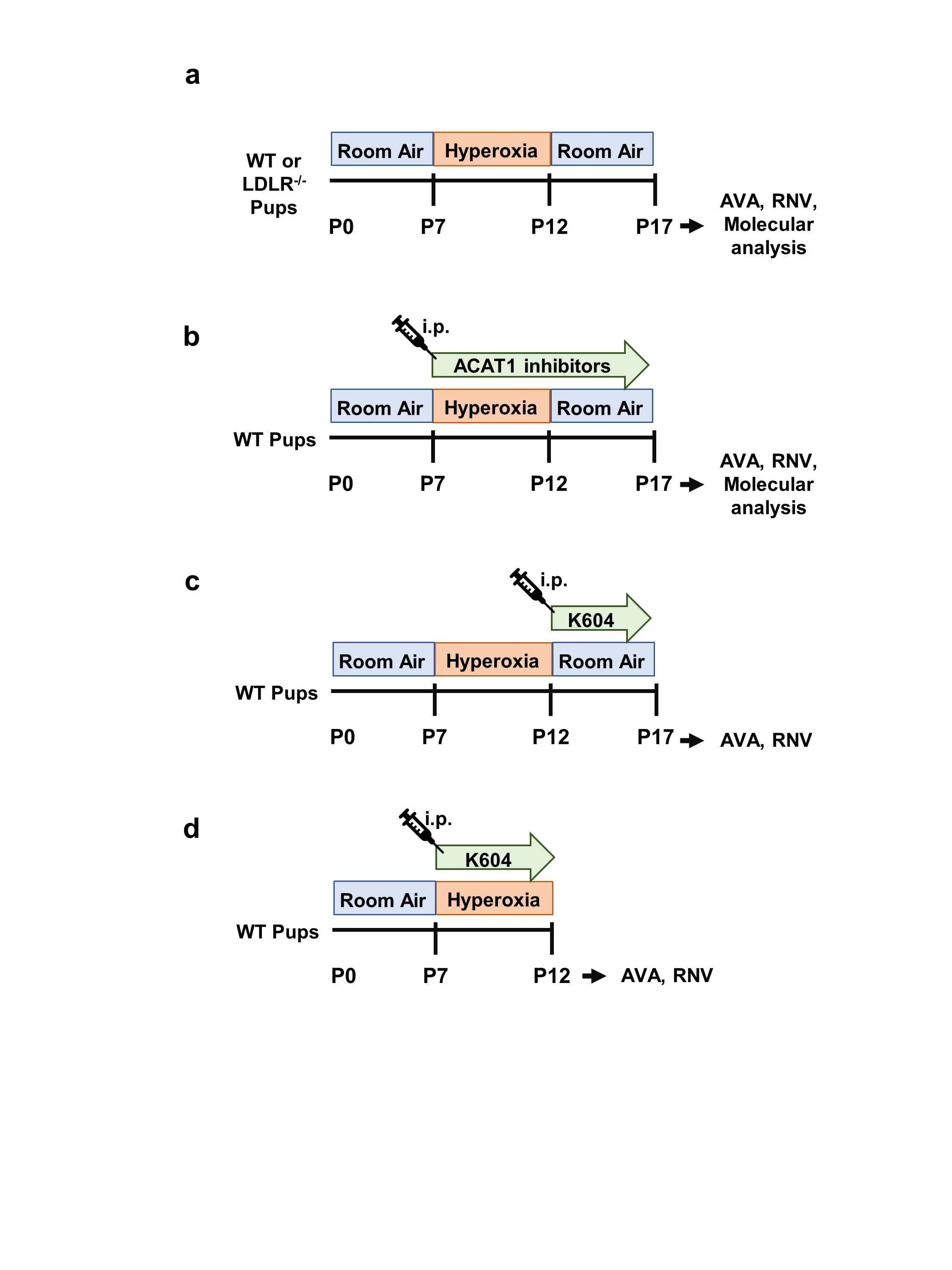

Supplement: Supplementary file 1 — Additional file 1: Figure S1. Schematic representation of experimental groups, treatments, and timepoints for analyses. a WT and LDLR−/− pups were subjected to OIR at P7 and sacrificed on P17. b–d WT pups were treated with ACAT inhibitor or vehicle from b P7 to P16 and sacrificed on P17 or c P12 to P16 and sacrificed on P17. d WT pups were treated with ACAT inhibitor or vehicle from P7 to P11 and sacrificed on P12. [file 12974_2023_2700_MOESM1_ESM.tif]

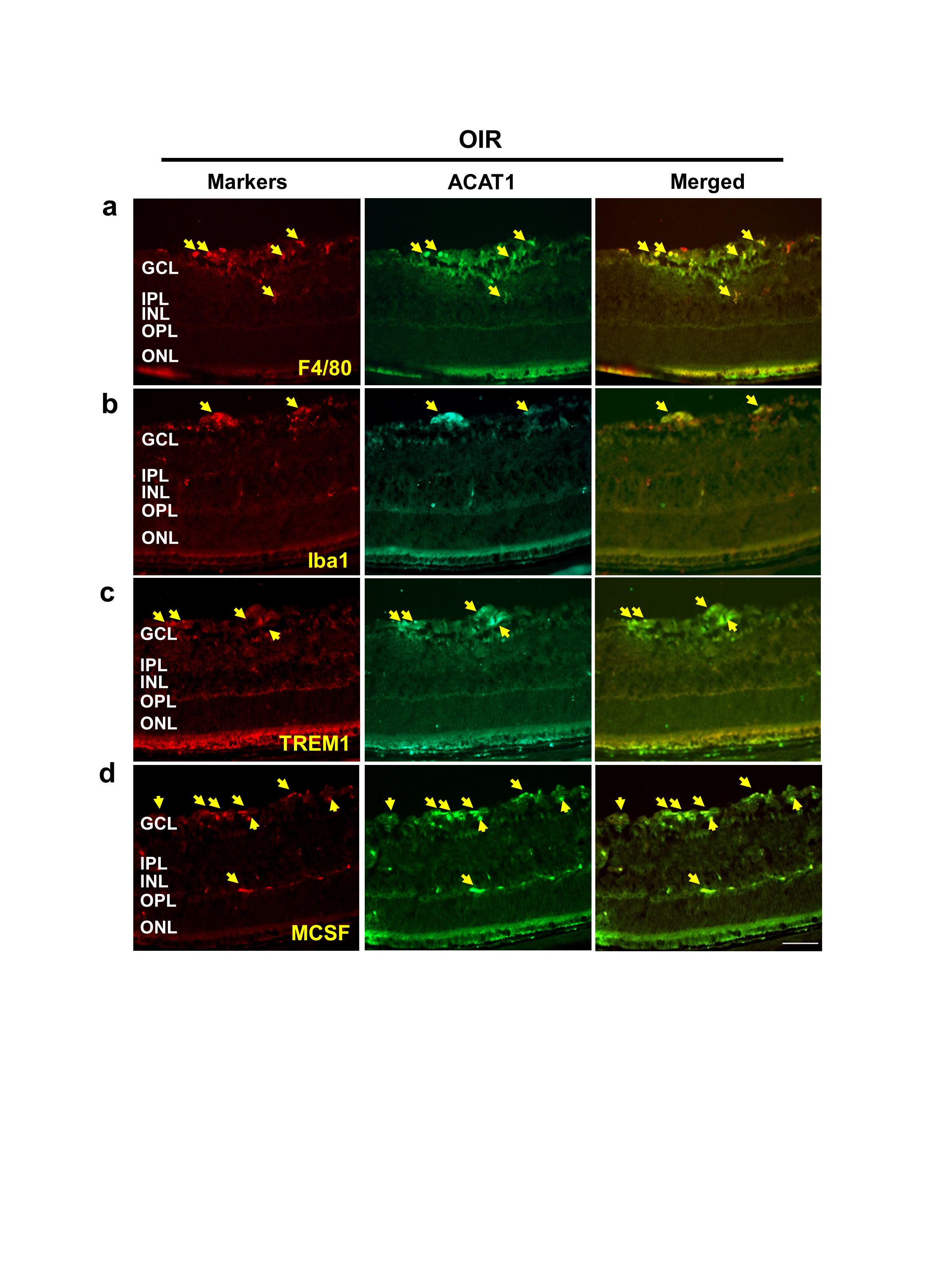

Supplement: Supplementary file 2 — Additional file 2: Figure S2. ACAT1 expression is colocalized with F4/80, Iba1, TREM1, and MCSF in OIR retinas. Mice were maintained in hyperoxia from P7 to P12 and then returned to room air. Eyes were enucleated at P17 and frozen sections were prepared for immunofluorescence imaging. ACAT1 is colocalized with a F4/80, b Iba1, c TREM1, d MCSF in areas of RNV. The areas of colocalization are indicated by yellow arrows. n = 4–6, scale bar = 60 μm. [file 12974_2023_2700_MOESM2_ESM.tif]

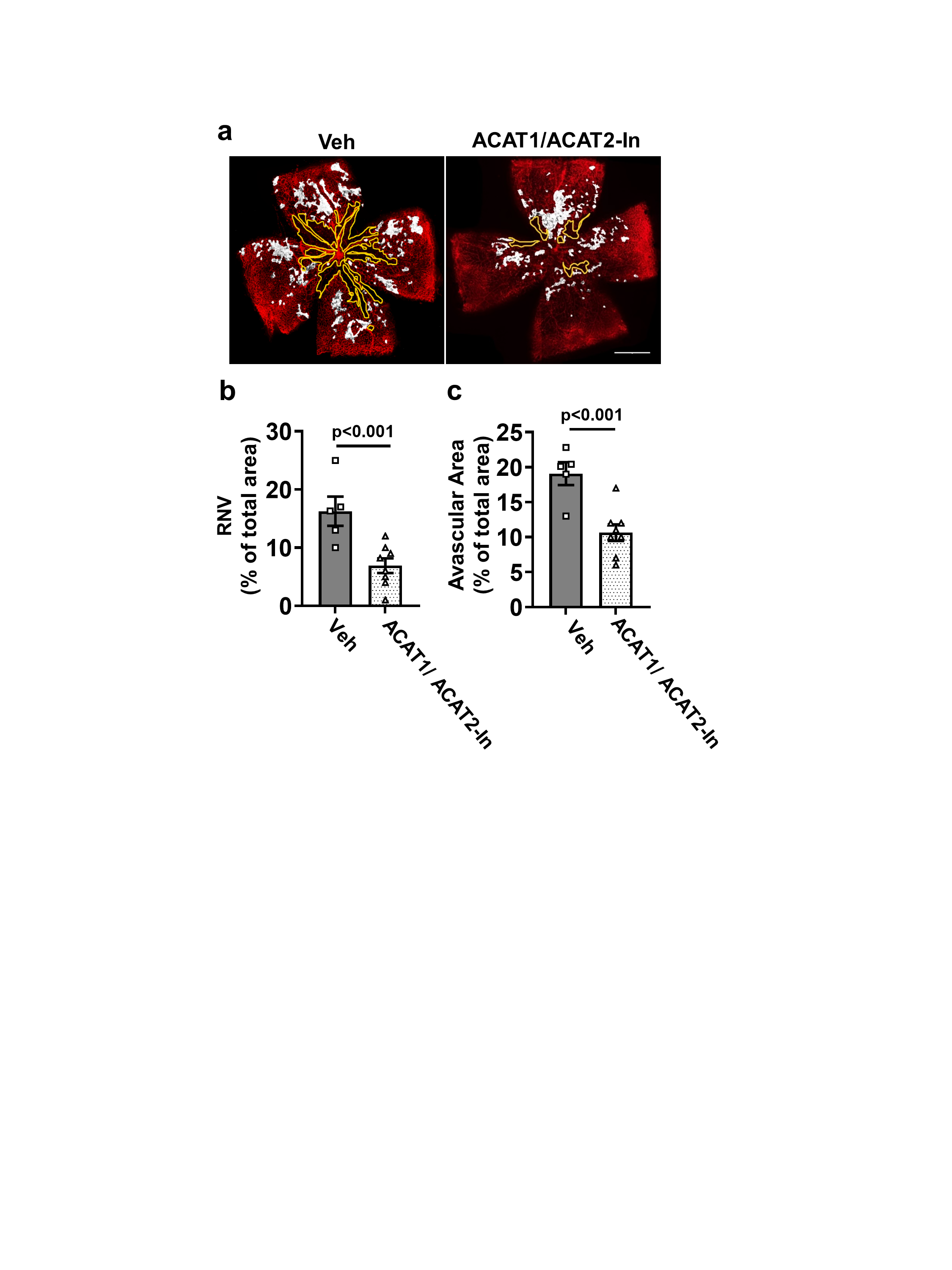

Supplement: Supplementary file 3 — Additional file 3: Figure S3. Inhibition of ACAT1/ACAT2 decreases RNV and AVA area in OIR retinas. OIR mice were treated with the ACAT1/ACAT2 inhibitor or vehicle from P7 to P16. Eyes were enucleated at P17 and prepared for retina flatmount analysis. a Retinal vessels were visualized by IB4 labeling and RNV (white highlighted areas) and AVA (yellow outline) were quantified. b, c Treatment with ACAT1/ACAT2 inhibitor significantly reduced the formation of RNV and decreased the AVA. n = 5–9, scale bar = 300 μm. [file 12974_2023_2700_MOESM3_ESM.tif]

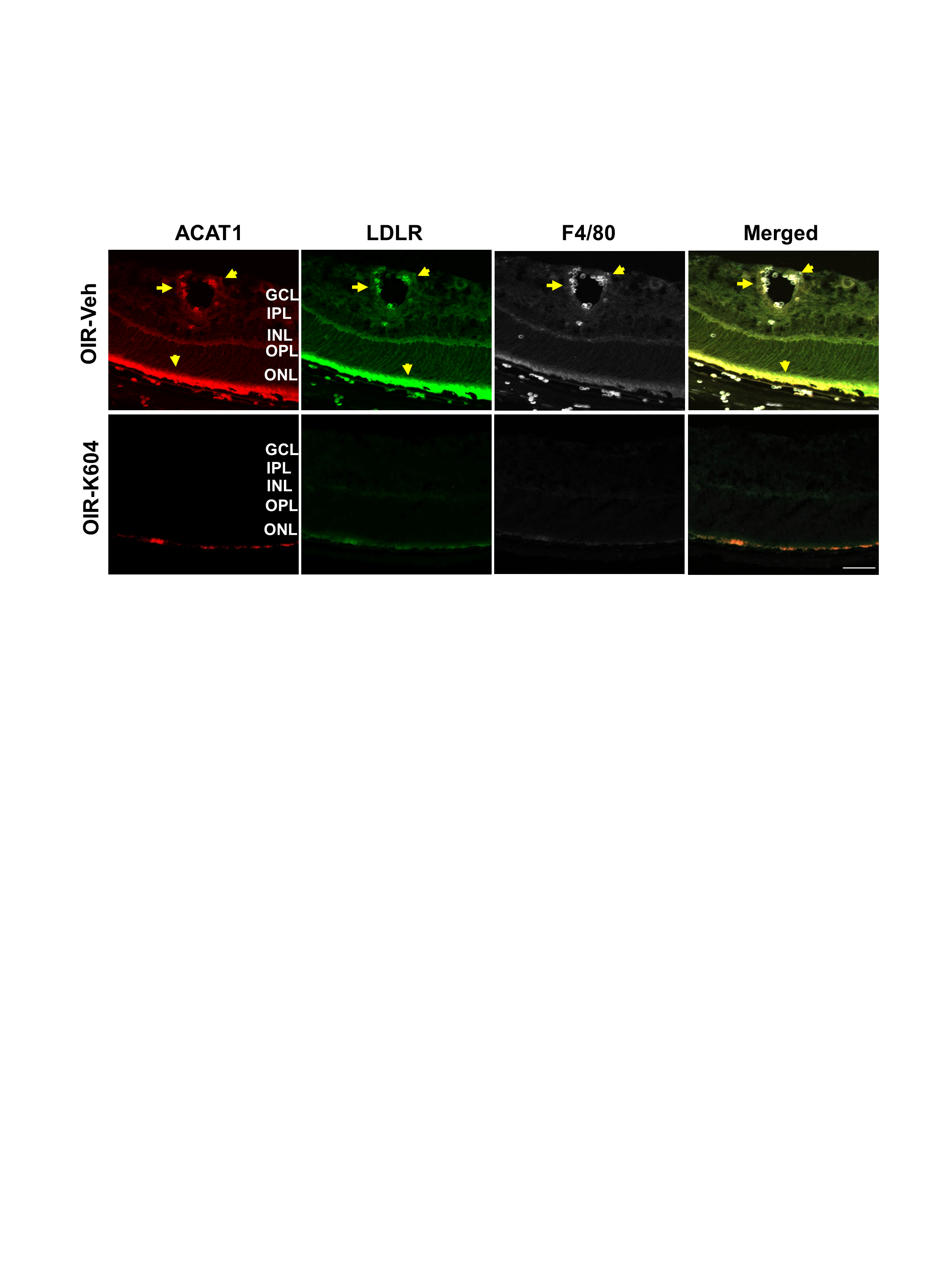

Supplement: Supplementary file 4 — Additional file 4: Figure S4. ACAT1 inhibition suppresses OIR-induced increase in ACAT1, LDLR, and F4/80-positive perivascular macrophage/microglia. OIR mice were treated with K604 or vehicle from P7 to P16 and frozen sections were prepared for immunofluorescence imaging. K604 treatment suppressed the OIR-induced increase in ACAT1, LDLR, and F4/80-positive perivascular macrophage/microglia. n = 4, scale bar = 40 μm. [file 12974_2023_2700_MOESM4_ESM.tif]

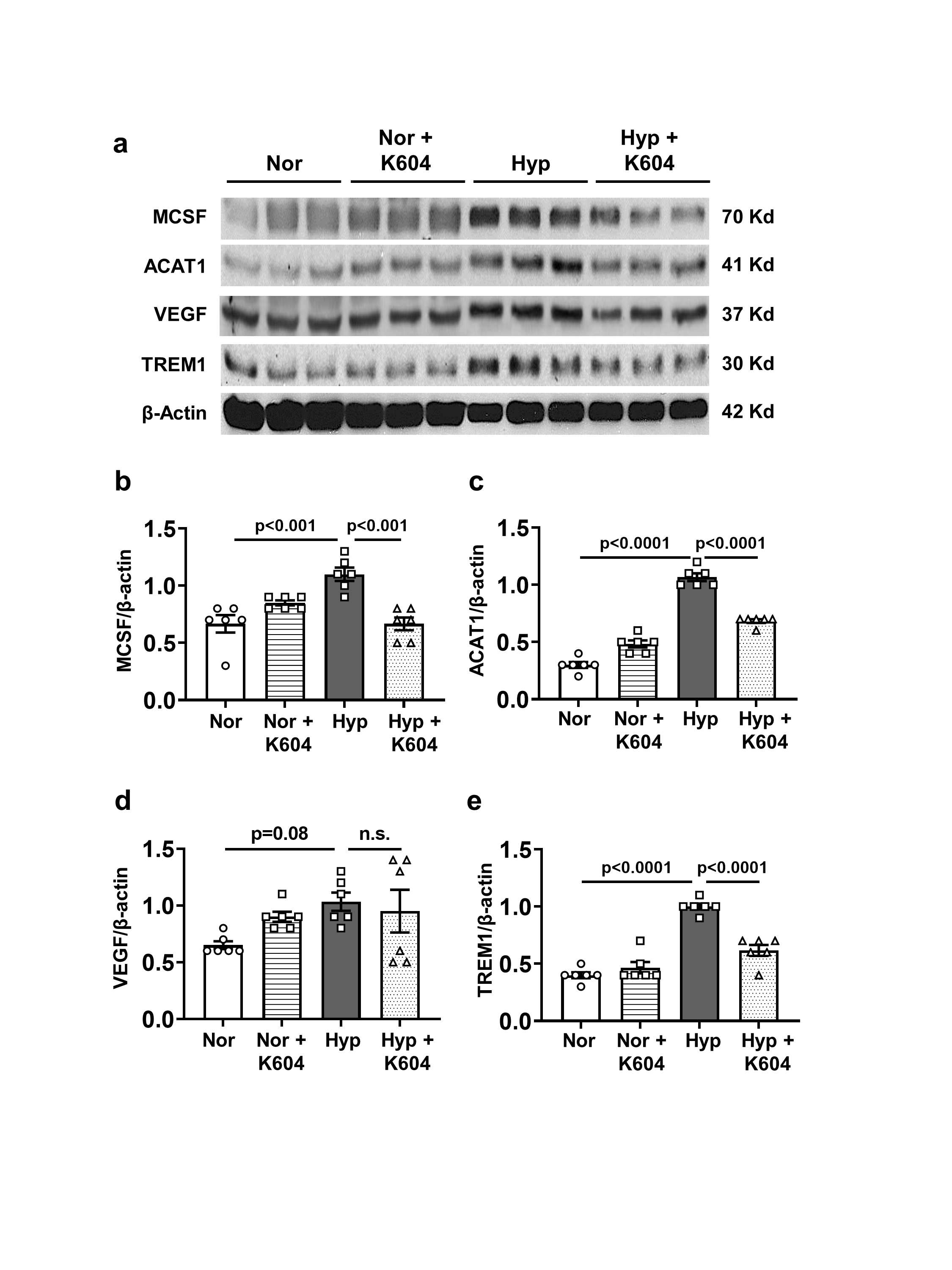

Supplement: Supplementary file 5 — Additional file 5: Figure S5. Inhibiting ACAT1 attenuates hypoxia-induced increases in expression of ACAT1 and inflammatory mediators in THP1 macrophages. Human THP1 cells were cultured in RPMI medium containing 5 mM glucose and 2% FBS and subjected to hypoxia (1% O2) or normoxia (21% O2) with K604 or vehicle for 16 h. a Western blotting, and b–e quantification shows upregulation of MCSF (a, b), ACAT1 (a, c), and TREM1 (a, e) after hypoxia treatment as compared with normoxia. K604 treatment markedly suppressed all except VEGF (a, d). Mean ± SEM, n = 6. [file 12974_2023_2700_MOESM5_ESM.tif]

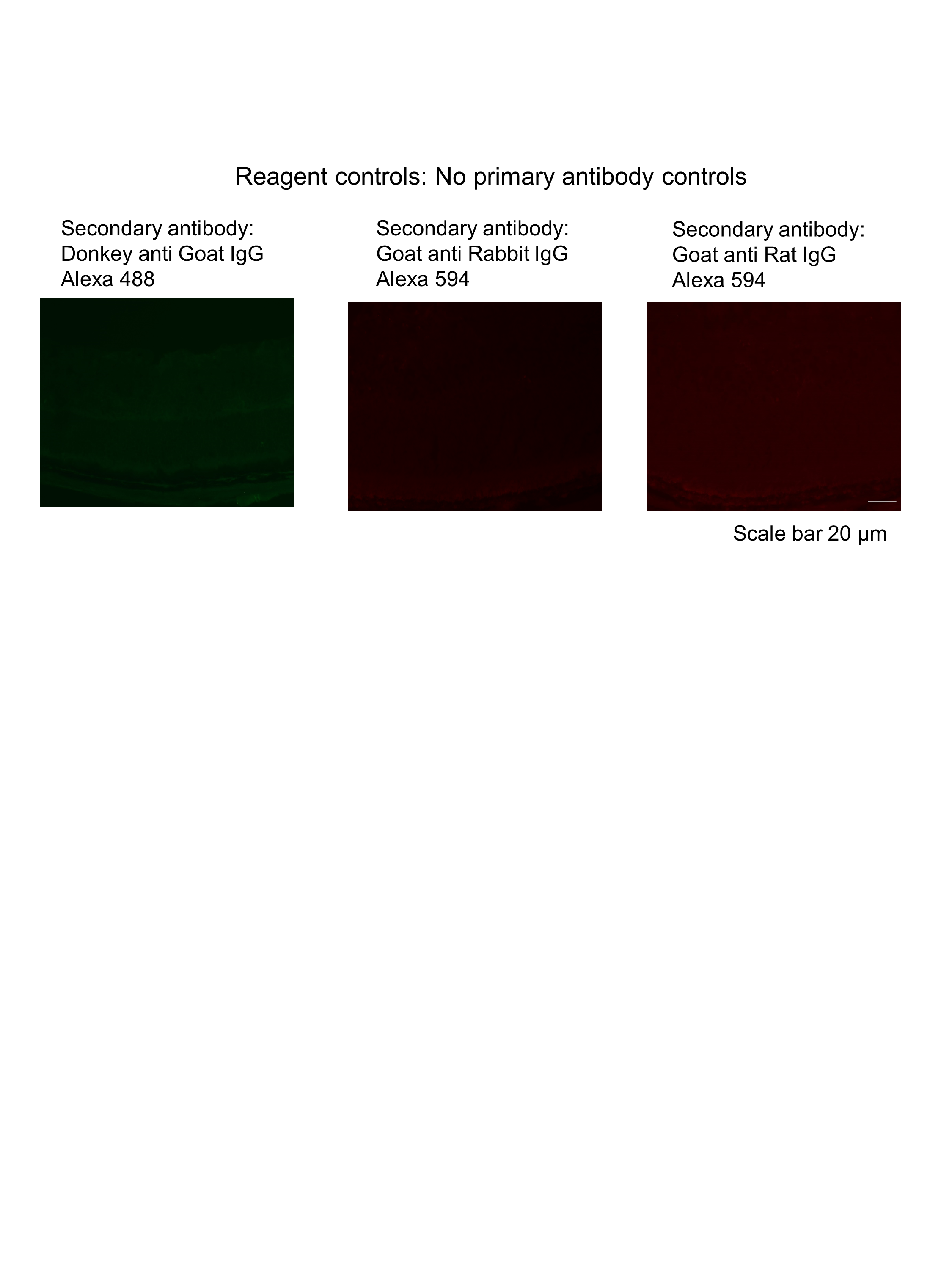

Supplement: Supplementary file 6 — Additional file 6: Figure S6. Images of no primary antibody controls. [file 12974_2023_2700_MOESM6_ESM.tif]

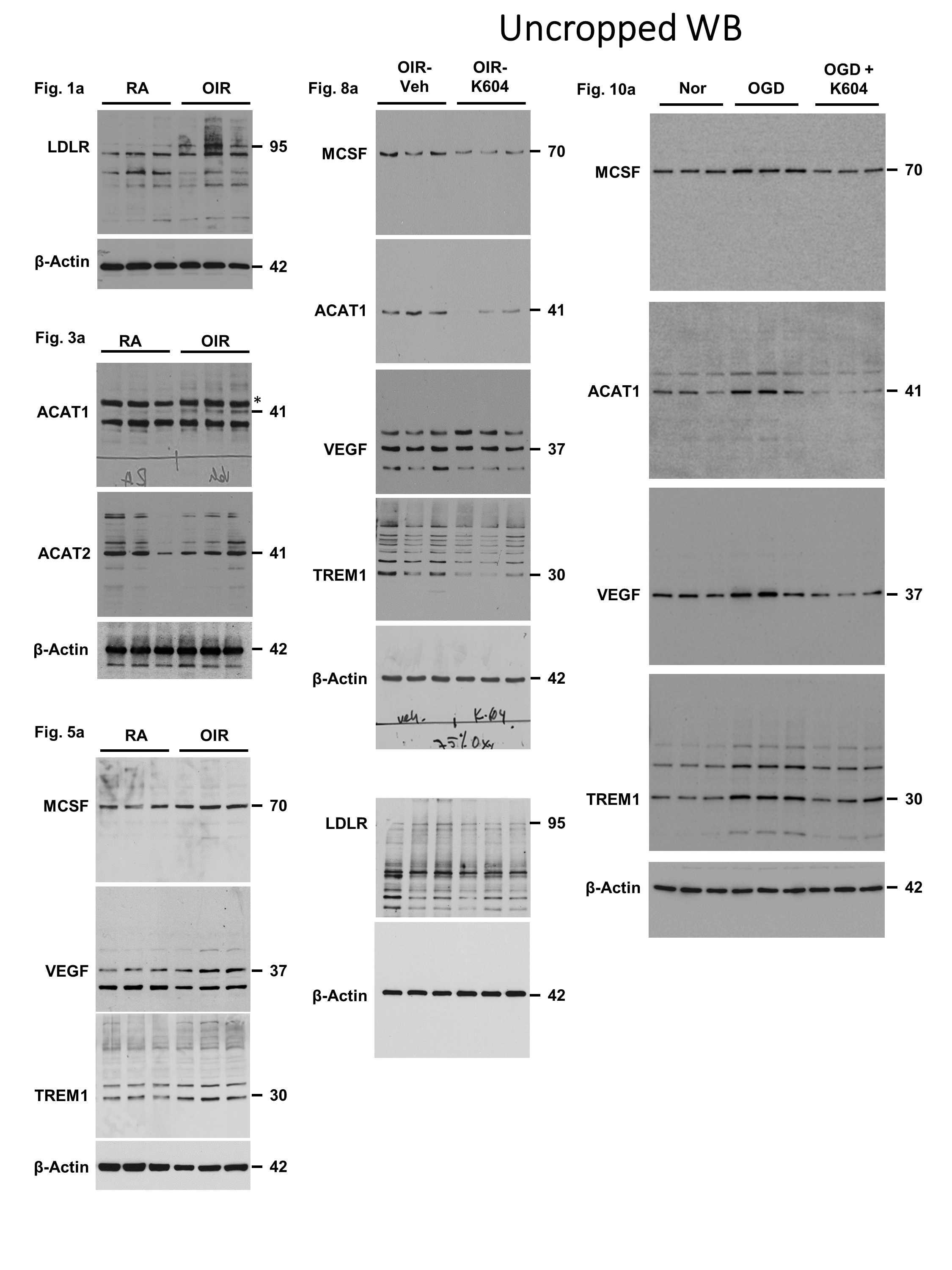

Supplement: Supplementary file 7 — Additional file 7: Figure S7. Uncropped images of Western blots. [file 12974_2023_2700_MOESM7_ESM.tif]

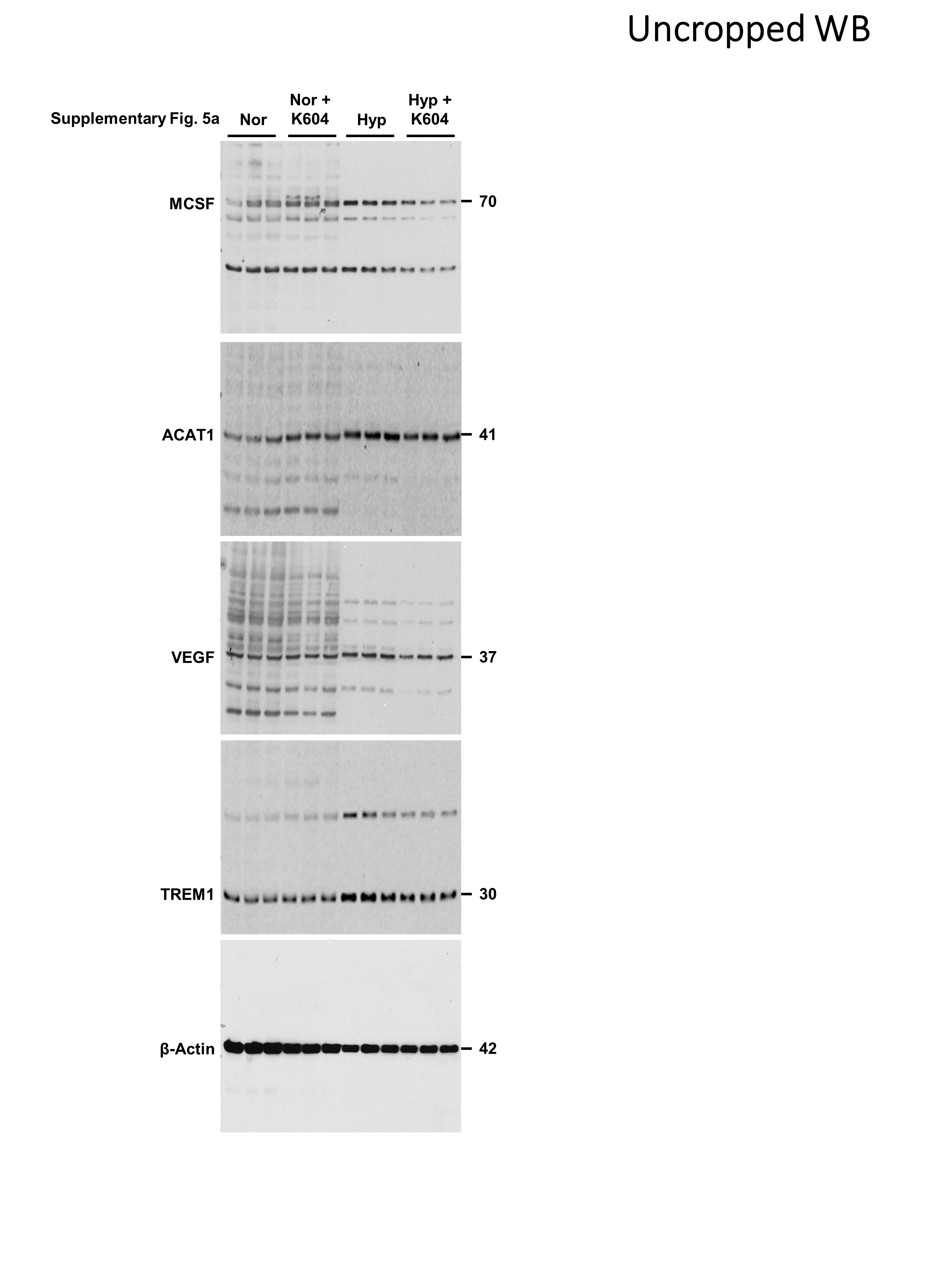

Supplement: Supplementary file 8 — Additional file 8: Figure S8. Uncropped images of Western blots. [file 12974_2023_2700_MOESM8_ESM.tif]
