# Supplementary material for: Role of acyl-coenzyme A: cholesterol transferase 1 (ACAT1) in retinal neovascularization
Source: J Neuroinflammation. 2023 Jan 23;20:14. doi: 10.1186/s12974-023-02700-5 (PMC9869542; doi:10.1186/s12974-023-02700-5)
Supplement: Supplementary file 9 — Additional file 9: Table S1. List of reagents used in this study. [file 12974_2023_2700_MOESM9_ESM.docx]

**Table S1: List of reagents used in this study**

| **Reagent or Resource** | **Source** | **Catalog** | **Comments** |
| --- | --- | --- | --- |
| **Antibodies** | | | |
| Rabbit anti-M-CSF, polyclonal | Abcam | ab99178 | WB (1:1000); IHC (1:200) |
| Rabbit anti-TREM1, polyclonal | Proteintech | 11791-1-AP | WB (1:1000); IHC (1:200) |
| Goat anti-ACAT1, polyclonal | Novus Biologicals | NBP1-46140 | WB (1:1000); IHC (1:200) |
| Rabbit anti-VEGFA, polyclonal | Abcam | ab46154 | WB (1:1000) |
| Rat anti-F4/80, monoclonal | Abcam | ab6640 | IHC (1:200) |
| Rabbit anti-LDL Receptor, polyclonal | Abcam | ab30532 | WB (1:500); IHC (1:200) |
| Rabbit anti-Iba1 | WAKO Chemicals | 019-19741 | IHC (1:200) |
| Mouse anti-β-Actin, monoclonal | Sigma Aldrich | A1978 | WB (1:1000) |
| Rabbit anti-ACAT2 polyclonal | Proteintech | 14755-1-AP | WB (1:1000) |
| Amersham ECL mouse IgG, HRP-linked | Cytiva | NA931-100UL | WB (1:2000) |
| Amersham ECL rabbit IgG, HRP-linked | Cytiva | NA934-100UL | WB (1:2000) |
| Peroxidase AffiniPure donkey anti-goat IgG (H+L) | Jackson Immunoresearch | 705-035-147 | IHC (1:400 to 1:600) |
| Donkey anti-rat IgG (H+L) Alexa Fluor 594 | Invitrogen | A21209 | IHC (1:400 to 1:600) |
| Donkey anti-rat IgG (H+L) Alexa Fluor 647 | Invitrogen | A48272 | IHC (1:400 to1:600) |
| Donkey anti-goat IgG (H+L) Alexa Fluor 488 | Invitrogen | A11055 | IHC (1:400 to1:600) |
| Donkey anti-goat IgG (H+L) Alexa Fluor 594 | Invitrogen | A11058 | IHC (1:400 to1:600) |
| Donkey anti-rabbit IgG (H+L) Alexa Fluor 488 | Invitrogen | A21206 | IHC (1:400 t0 1:600) |
| Donkey anti-rabbit IgG (H+L) Alexa Fluor 594 | Invitrogen | A21207 | IHC (1:400 to 1:600) |
| Donkey anti-rabbit IgG (H+L) Alexa Fluor 647 | Invitrogen | A31573 | IHC (1:400 to 1:600) |
| **Chemicals and Recombinant Proteins** | | | |
| Isolectin GS-IB4, Alexa Fluor 594 | Thermofisher Scientific | I21413 | IHC (1:200) |
| N-[3-(4-hydroxyphenyl)-1-oxo-2-propenyl]-L-phenylalanine, methyl ester | Santa Cruz | sc-221976A | ACAT1/ACAT2 inhibitor |
| K-604 dihydrochloride | Millipore Sigma | SML1837 | ACAT1 inhibitor |
| K-604 dihydrochloride | MedChemExpress LLC | HY-100400A | ACAT1 inhibitor |
| Filipin III | Sigma Aldrich | F4767 |  |
| Oil Red O | Sigma Aldrich | O9755 |  |
| Fixative Solution (4% formaldehyde, methanol-free), 5x20ml | Thermofisher Scientific | R37814 |  |
| Mounting Medium | Vector Labs | H-1000 |  |
| Matrigel, GFR-Basement Membrane Matrix | Corning | 354230 |  |
| VEGF, recombinant human | BRB Preclinical Repository, NCI | 32201 |  |
| **Commercial Kits** | | | |
| Cholesterol/Cholesterol Ester-Glo(TM) Assay | Promega | J3190 |  |
| RNAqueous™-4PCR Total RNA Isolation Kit | Invitrogen | AM1914 |  |
| TaqMan™ Universal PCR Master Mix | Invitrogen | 4324018 |  |
| Power SYBR™ Green PCR Master Mix | Invitrogen | 4367659 |  |
