# Supplementary material for: Role of acyl-coenzyme A: cholesterol transferase 1 (ACAT1) in retinal neovascularization
Source: J Neuroinflammation. 2023 Jan 23;20:14. doi: 10.1186/s12974-023-02700-5 (PMC9869542; doi:10.1186/s12974-023-02700-5)
Supplement: Supplementary file 10 — Additional file 10: Table S2. Primer sequences used in qRT-PCR. [file 12974_2023_2700_MOESM10_ESM.docx]

**Table S2: Primer Sequences Used in qRT-PCR**

| **Mouse Gene** | **Sequence / Reference ID** | **Probes** | **Source** |
| --- | --- | --- | --- |
| *Hprt* | Forward: GAAAGACTTGCTCGAGATGTCATG Reverse: CACACAGAGGGCCACAATGT | SYBR Green | IDT |
| *IL-1β* | Forward: TGCCACCTTTTGACAGTGATG Reverse: ATGTGCTGCTGCGAGATTTG | SYBR Green | IDT |
| *Tnf* | Forward: GGTCCCCAAAGGGATGAGAA Reverse: TGAGGGTCTGGGCCATAGAA | SYBR Green | IDT |
| *Acat1* | Forward: GCAGGGAAGTTTGCCAGTGAGA  Reverse: GAACACGGTCTTGAGCTTTGGC | SYBR Green | IDT |
| *Trem1* | Forward: CCTGTTGTGCTCTTCCATCCTG  Reverse: CGGGTTGTAGTTGTGTCACTGG | SYBR Green | IDT |
| *Mcsf* | Forward: GCCTCCTGTTCTACAAGTGGAAG  Reverse: ACTGGCAGTTCCACCTGTCTGT | SYBR Green | IDT |
| *Tyrobp (Dap12)* | Forward: GTGACTTGGTGTTGACTCTGCTG  Reverse: GATAAGGCGACTCAGTCTCAGC | SYBR Green | IDT |
| *HprtT* | Mm00446968-m1 | FAM | TaqMan |
| *IL6* | Mm 00446190-m1 | FAM | TaqMan |
